# Quantitative color fundus photography parameters as potential biomarkers of axial length progression: evidence from a machine learning cohort study

**DOI:** 10.3389/fcell.2026.1753213

**Published:** 2026-01-26

**Authors:** Zixun Wang, Feifei Han, Xiaoling Zhang, Jingjie Ding, Jingtao Yu, Xueshuo Xie, Zhiqing Li, Bei Du, Ruihua Wei

**Affiliations:** 1 Tianjin Key Laboratory of Retinal Functions and Diseases, Tianjin Branch of National Clinical Research Center for Ocular Disease, Eye Institute and School of Optometry, Tianjin Medical University Eye Hospital, Tianjin, China; 2 Handan Eye Hospital (The Third Hospital of Handan), Handan, Hebei, China; 3 Haihe Lab of ITAI, Naikai University, Tianjin, China

**Keywords:** axial length progression, color fundus photography, machine learning, pediatric myopia, retinal microvasculature, shap

## Abstract

**Purpose:**

Early identification of children at risk for accelerated axial elongation is essential for implementing timely myopia control strategies. Quantitative parameters derived from color fundus photography (CFP) may capture subtle structural and microvascular features relevant to axial length (AL) progression, yet their predictive value remains insufficiently characterized. To develop and validate a machine learning–based model integrating CFP-derived quantitative biomarkers and clinical characteristics to predict 1-year AL progression in school-aged children.

**Methods:**

This cohort study included 693 children aged 6–10 years from Tianjin, China. AL progression >0.2 mm over 1 year was defined as significant elongation. Baseline clinical variables and 144 quantitative CFP metrics were evaluated. Feature selection was performed using Least Absolute Shrinkage and Selection Operator (LASSO) regression, logistic regression screening, and expert ophthalmologic assessment. Seven machine learning algorithms were developed using fivefold cross-validation, with hyperparameters optimized by grid search. Model performance was evaluated on an independent validation set using the area under the receiver operating characteristic (ROC) curve (AUC), F1 score, and other metrics. The best-performing model was interpreted using Shapley Additive Explanations (SHAP) and Local Interpretable Model-Agnostic Explanations (lime).

**Results:**

Of the 693 included children, 457 (65.9%) exhibited AL progression >0.2 mm. LASSO regression selected 39 candidate variables, and 12 predictors were ultimately incorporated into the model construction. Among all algorithms, the Random Forest (RF) model achieved the best discrimination, with an AUC of 0.961 (95% CI: 0.933–0.984) and the highest F1 score. Decision curve analysis (DCA) demonstrated a favorable net benefit across clinically relevant thresholds. SHAP analysis indicated that retinal venous density, venous fractal dimension, presence of leopard-spot lesions, vascular fractal dimension, and inferior-region vascular density were among the most influential predictors of AL progression.

**Conclusion:**

The RF model, which combines clinical characteristics with CFP-derived quantitative biomarkers, accurately predicts short-term AL progression in children. Retinal microvascular and fundus structural parameters significantly contributed to model performance, underscoring their potential as early indicators of myopic AL elongation.

## Introduction

Myopia among children is a serious public health issue (Burton et al., 2021). By 2050, approximately 50% of the global population is expected to be nearsighted, with 10% suffering from high myopia (HM). This trend continues to grow (Holden et al., 2016; Pan et al., 2025). To address this public health challenge, early detection of children with myopia or pre-myopia is crucial for preventing the progression of myopia during childhood (Zhang S. et al., 2025; Cai et al., 2025). The assessment of axial length (AL) serves not only as a critical parameter for myopia progression but also explains the ocular elongation and biomechanical alterations associated with myopia, ultimately leading to irreversible pathological changes in the fundus of the eye (Cai et al., 2025; Liu et al., 2025; Tao et al., 2025). Currently, there are no biological markers for the progression of AL in early childhood myopia.

As the AL increases, changes in the shape of the eyeball also alter the fundus (Sui et al., 2024). In recent years, color fundus photography (CFP) has also played a role in detecting HM fundus changes (Zhu et al., 2022; Hayashi et al., 2010). Currently, CFP remains the diagnostic basis for retinal lesions in ophthalmic diagnosis and treatment. However, in the early stages of myopia, no retinal lesions are detected, and CFP appears indistinguishable from normal CFP. Recent studies have identified an association between the emergence of leopard spots and the progression of childhood myopia (Gong et al., 2024; Sameshima et al., 2024). Nevertheless, similar CFP biological parameters still fail to indicate the risk of early childhood AL progression adequately.

With the advancement of deep learning (DL) and machine learning (ML), artificial intelligence (AI) has demonstrated significant advantages in image recognition and disease prediction (Hu M. et al., 2025; Yii et al., 2025). Previous studies have utilized CFP in elderly individuals to predict AL length, showing a correlation between CFP and AL (Wang et al., 2024; Oh et al., 2023; Yang et al., 2024). Wang et al. investigated Sphere Equivalent (SE) and its progression using CFP (Wang Z. et al., 2025; He et al., 2024). Quantified CFP using segmentation technology provides more biologically interpretable parameters (He et al., 2024; Chen et al., 2024). We previously employed quantitative indicators from the Children’s CFP to perform binary classification prediction of myopia risk (Zhang X. et al., 2025). However, the predictive value of CFP in childhood AL growth has not been studied.

Therefore, this study aims to establish a predictive model for AL progression using quantitative baseline CFP and ocular parameters, and to conduct an interpretability study of the parameters incorporated into the model via artificial intelligence to identify potential biomarkers indicating AL growth in school-aged children.

## Methods

### Data collection

**FIGURE 1:**
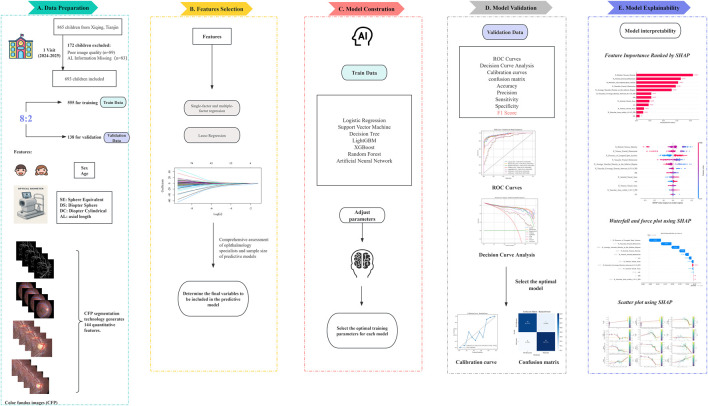
The overall flowchart of the study, including data collection and data types, training and validation set partitioning **(A)**, feature selection for predictive models **(B)**, model construction and optimal parameter selection **(C)**, performance comparison and evaluation of predictive models **(D)**, and interpretability analysis of predictive models **(E)**.

The study was approved by the Ethics Committee of Tianjin Medical University Eye Hospital and conducted in accordance with the principles outlined in the Declaration of Helsinki (2024KY-67). This cohort study was conducted in a randomly selected elementary school in Xi Qing District, Tianjin, from April 2024 to October 2025. Children aged 6–10 years were included in the study. Children were excluded from the study if their parents or legal guardians did not provide consent for their participation. Children with systemic diseases (e.g., congenital heart disease, ocular trauma, or ophthalmic diseases such as glaucoma, cataracts, infectious eye infections, and strabismus) were also excluded. A total of 865 children were included in the study. We defined 1-year AL progression ≤0.2 mm as the AL nonprogressive group and AL progression >0.2 mm as the myopic AL progressive group (Lanca et al., 2021; Li et al., 2023). No standardized myopia control interventions were implemented as part of the study protocol. All children underwent a complete ophthalmic examination, including slit-lamp biomicroscope, best-corrected visual acuity (BCVA), Intraocular pressure (IOP) (CT-1, Topcon, Japan), AL (Lenstar LS-900, Haag-Streit AG, Switzerland), and SER (KR-800, Topcon, Japan). All CFP images were examined using a 45° fundus camera (Canon Inc., 9-1 Kanagawa, Japan). Due to poor photo quality and loss to follow-up, 693 children were ultimately included in the study. To avoid bilateral correlation, this study included only one CFP per child for analysis (Hu X. et al., 2025). All biometric and refractive measurements corresponded to the same eye as the included CFP image. The research design process is shown in Figure 1.

### Generation and selection of CFP-based indicators

Briefly, CFP images were acquired using a standardized protocol, followed by automated object detection and semantic segmentation to identify retinal structures. Quantitative metrics were subsequently derived from segmented outputs and reviewed by experienced ophthalmologists. Object detection and semantic segmentation models were applied to identify and quantify key retinal characteristics, including tessellation patterns, optic disc and cup morphology, parapapillary atrophy, and vascular features. Image labeling followed a semi-automated, machine-assisted pipeline. Two board-certified ophthalmologists (each with at least 5 years of experience in fundus evaluation) independently reviewed and refined the annotations. The first clinician conducted preliminary edits, while the second, a senior reviewer with over 8 years of experience, provided final adjudication. Owing to their greater expertise, the adjudicator’s markings served as the reference standard. Quantitative disclosure techniques were detailed in our previous research (Zhang X. et al., 2025). Additional methodological details are available in Document S1.

This study included baseline information such as sex, age, cycloplegic SE, Diopter Sphere (DS), and AL. We obtained 144 quantitative indicators of CFP. The quantitative CFP variables were categorized into the following areas: (1) the presence of leopard spots and atrophic arcs; (2) vascular-related parameters, including the length, area, and angle of arterioles and venules, and vascular density (VD) and parameters in a particular area; (3) optic disc-related parameters, including morphologic quantification, relative optic disc position of the optic disc, and optic cup ratio of the optic disc; (4) the distance of essential locations in the fundus (macula, optic disc, and vascular arch). In addition to the quantitative metrics of CFP segmentation itself, there are quantitative metrics representing the regularity and curvature of the vessels, calculated using higher-order mathematics. For example, the vascular fractal dimension (VFD) is computed using the box-counting principle: cover the target area with square boxes of different sizes and count the number of boxes that cover at least part of the target area for each box size (Prasad, 2025). The smaller the box, the higher the number. A linear fit is made between the logarithm of the box size and the logarithm of the number of boxes, and the slope is the fractal dimension. Vessel density in each area is calculated as the area of vessels in that area/the overall area of the fundus (Guan et al., 2025). Retinal vascular curvature was defined as the integral of the squared curvature of the retinal vascular pathway, normalized by the total path length (Sekiryu et al., 2025). Additional methodological details are available in Document S1.

The sample data (subjects) were divided into a training set and a validation set (training set: validation set = 8:2). Due to the large number of variables after image quantization, the Least Absolute Shrinkage and Selection Operator (LASSO) was used to select features, which is a method to introduce L1 regularization, select features, and reduce dimensions by compressing coefficients, screening features with significant contributions, and eliminating redundant features (Song et al., 2026). We also employ single-factor and multiple-factor regression for variable screening. The final determination of predictive factors was based on the clinical selection of comprehensive ophthalmology specialists and the sample size and number of included predictors in the clinical prediction model (Riley et al., 2019).

### Model development and validation

Model training is conducted on the designated training set, while model validation is performed on a separate, independent validation set. The two processes are mutually independent. Seven machine learning algorithms—extreme gradient boosting (XGBoost), support vector machine (SVM), Random Forest (RF), artificial neural network (ANN), decision tree, logistic regression (LR), and light gradient boosting machine (LightGBM)—were implemented to develop the predictive models. Model robustness was assessed using fivefold cross-validation. Hyperparameters for each algorithm were tuned via grid search conducted exclusively within the training folds to avoid information leakage. The configuration yielding the highest area under the receiver operating characteristic curve (AUC) was selected as the optimal model. For performance estimation, 95% confidence intervals for AUC were calculated in the validation cohort using 1,000 bootstrap resamples. The predictive models were trained on the designated training dataset, and the optimal model was subsequently evaluated on the validation cohort.

Model performance was quantified using the AUC, sensitivity, specificity, F1 score, recall, accuracy, Kappa, and Youden’s J. To further assess the clinical applicability of the models, decision curve analysis (DCA) and calibration curves were generated. DCA evaluates the net clinical benefit of a predictive model across a range of threshold probabilities, thereby demonstrating its value in guiding clinical decisions (Chalkou et al., 2023). We ultimately selected the optimal model by comprehensively evaluating both F1 scores and AUC. After choosing the optimal model, further examine its confusion matrix and calibration curve on the validation set. The calibration curve demonstrates the degree of agreement between predicted probabilities and actual outcomes, serving as an indicator for assessing model reliability and goodness-of-fit (Van Calster et al., 2019).

### Model interpretation

Shapley Additive Explanations (SHAP) values were calculated to assess the magnitude and direction of each variable’s influence on the model’s final classification. Features with larger absolute SHAP values were interpreted as having a more substantial contribution to predictive performance. These importance metrics were used to elucidate the behavior of the optimal model (Bhandari et al., 2022). To complement the global explanation provided by SHAP, the Local Interpretable Model-Agnostic Explanations (lime) method was further applied, offering case-level insights into how specific predictions were generated (Zhao et al., 2025; Huang et al., 2024; Guan et al., 2024). The SHAP dependence plot demonstrates the effect of a single feature on the output of the best model. Model training was conducted using Python 3.4 (https://www.python.org).

### Statistical analysis

Group differences were evaluated using Student’s t-test for normally distributed variables and the Wilcoxon rank-sum test for non-normal data. Distributional assumptions were assessed with the Kolmogorov–Smirnov or Shapiro–Wilk tests. Categorical variables were compared using the chi-squared or Fisher’s exact test, as appropriate. Univariable logistic regression was first applied to all candidate predictors, and variables with *p* < 0.05 were entered into a stepwise multivariable logistic regression to derive the final model. Continuous data are reported as mean ± standard deviation, and statistical significance was defined as p < 0.05 (two-tailed). Normally distributed continuous variables are presented as mean ± standard deviation, whereas non-normally distributed variables are summarized as median [interquartile range]. Distributional assumptions were assessed using the Shapiro–Wilk or Kolmogorov–Smirnov tests. All analyses were conducted in R (v4.4.2).

## Results

A total of 693 children were included in the analysis, of whom 236 (34.1%) were classified in the AL ≤ 0.2 mm group and 457 (65.9%) in the AL > 0.2 mm group. Baseline characteristics are summarized in Table 1; Supplementary Table S2.

**TABLE 1:** Baseline Information Form in this study.

Name	Level	Overall	ΔAL ≤0.2 mm	ΔAL >0.2 mm	p
n	​	693	236	457	​
Sex (%)	Male	345 (49.78)	139 (58.90)	206 (45.08)	0.001
​	Female	348 (50.22)	97 (41.10)	251 (54.92)	​
DS (median [IQR])	​	0.75 [0.00, 1.25]	1.00 [0.50, 1.50]	0.50 [0.00, 1.00]	<0.001
AL (median [IQR])	​	23.15 [22.66, 23.70]	23.08 [22.59, 23.62]	23.21 [22.69, 23.75]	0.043
SE (median [IQR])	​	0.38 [-0.25, 1.00]	0.75 [0.10, 1.25]	0.25 [-0.38, 0.88]	<0.001
Average vascular branching angle (median [IQR])	​	64.15 [59.61, 68.04]	65.10 [61.05, 68.77]	63.86 [59.07, 67.86]	0.048
Vascular fractal dimension (median [IQR])	​	1.54 [1.52, 1.56]	1.56 [1.54, 1.57]	1.53 [1.51, 1.55]	<0.001
Vascular fractal dimension in the inferior disk region (median [IQR])	​	1.35 [1.33, 1.38]	1.36 [1.34, 1.38]	1.35 [1.32, 1.37]	<0.001
Vascular density (median [IQR])	​	0.08 [0.07, 0.09]	0.09 [0.08, 0.10]	0.08 [0.07, 0.09]	<0.001
Vascular coverage density between 0.5-1.0 PD (median [IQR])	​	0.14 [0.13, 0.15]	0.14 [0.13, 0.15]	0.14 [0.12, 0.15]	<0.001
Vascular coverage density between 2.0-2.5 PD (median [IQR])	​	0.05 [0.04, 0.06]	0.06 [0.05, 0.06]	0.05 [0.04, 0.06]	0.016
Average vascular density within 3 mm of fovea centralis (median [IQR])	​	0.05 [0.04, 0.06]	0.05 [0.04, 0.06]	0.05 [0.04, 0.06]	0.047
Average vascular density in the inferior region (median [IQR])	​	0.11 [0.09, 0.13]	0.12 [0.11, 0.14]	0.10 [0.08, 0.12]	<0.001
Angle between line connecting fovea and optic disk.unit. Degrees. (Median [IQR])	​	4978.44 [4781.11, 5145.04]	5011.14 [4810.17, 5165.91]	4964.53 [4760.75, 5136.06]	0.048
Retinal arterial density (median [IQR])	​	0.04 [0.03, 0.04]	0.04 [0.03, 0.04]	0.04 [0.03, 0.04]	0.019
Retinal venous density (median [IQR])	​	0.05 [0.04, 0.05]	0.06 [0.05, 0.07]	0.05 [0.03, 0.05]	<0.001
Venous fractal dimension (median [IQR])	​	1.43 [1.40, 1.45]	1.45 [1.43, 1.47]	1.42 [1.38, 1.44]	<0.001
Average arterial branching angle (median [IQR])	​	64.97 [57.71, 70.77]	65.17 [58.74, 72.56]	64.77 [57.20, 69.78]	0.039
Arterial vessel area (median [IQR])	​	4.25 [3.66, 4.76]	4.29 [3.79, 4.81]	4.20 [3.59, 4.72]	0.049
Venous vessel area (median [IQR])	​	5.94 [5.37, 6.40]	6.23 [5.66, 6.67]	5.77 [5.16, 6.25]	<0.001
Vascular area (median [IQR])	​	10.19 [9.29, 10.89]	10.59 [9.81, 11.33]	9.93 [9.10, 10.76]	<0.001
Vascular area within 1.0–1.5 PD (mean (SD))	​	1.63 (0.31)	1.67 (0.32)	1.61 (0.30)	0.039
Vascular area within 2.0–2.5 PD (median [IQR])	​	1.37 [1.24, 1.54]	1.51 [1.33, 1.66]	1.32 [1.15, 1.47]	<0.001

The distribution of sex differed significantly between groups (*p* = 0.001), with a higher proportion of females in the AL > 0.2 mm group. The prevalence of atrophic regions did not differ between groups, whereas leopard spot lesions were more frequently observed in eyes with AL progression >0.2 mm (*p* < 0.001). Age did not differ significantly between the two groups.

Refractive and biometric parameters demonstrated several between-group differences. Children in the AL ≤ 0.2 mm group had higher spherical equivalent (*p* < 0.001) and DS values (*p* < 0.001), while baseline AL was slightly greater in the AL > 0.2 mm group (*p* = 0.043).

Multiple vascular morphological features differed between groups. Significant differences were noted in average vascular branching angle (*p* = 0.048), vascular fractal dimension (*p* < 0.001), inferior disk region fractal dimension (*p* < 0.001), vascular density (*p* < 0.001), vascular coverage density at 0.5–1.0 PD (*p* < 0.001), average vascular density within 3 mm of the fovea (*p* = 0.047, *p* < 0.05), inferior-region vascular density (*p* < 0.001), retinal arterial density (*p* = 0.019, *p* < 0.05), retinal venous density (*p* < 0.001), venous fractal dimension (*p* < 0.001), arterial branching angle (*p* = 0.039, *p* < 0.05), and venous and arterial vessel area (both *p* < 0.05). Several measures of vascular area across parafoveal regions also differed significantly, including values within 1.0–1.5 PD (*p* = 0.039, *p* < 0.05) and 2.0–2.5 PD (*p* < 0.001). In contrast, most optic disc and cup parameters—including optic disc area, diameters, tilt angle, rim distances, and cup–disc ratios—showed no significant differences between groups. Arterial and venous diameter measures, curvature metrics, and various regional vascular parameters also did not differ significantly.

**FIGURE 2:**
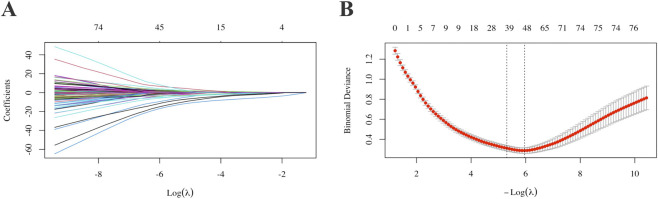
Selection of model variable parameters for AL progression. For each variable, parameter selection was performed using single-factor, multiple-factor regression and LASSO **(A,B)**. AL: Axial Length.

Table 2; Supplementary Table S3 present the results of univariate and multivariate regression analyses for the included variables. Following the multivariate regression analysis, six variables were considered for inclusion in the model. Figure 2 shows the variables and their quantities ultimately selected by the LASSO regression method. The LASSO model minimized binomial deviance when the log(λ) value reached the optimal penalty parameter (0.0037). LASSO selected 39 variables for inclusion in the model. After ophthalmologists determined the variables to be included based on variable screening results and comprehensive consideration of sample size and included variables, the following baseline information was ultimately incorporated: sex, DS, AL, and nine retinal quantitative parameters (Retinal Venous Density, Venous Fractal Dimension, Presence of Leopard Spot Lesions, Vascular Fractal Dimension, Average Vascular Density in the Inferior Region, Vascular Coverage Density between 0.5-1.0 PD, Arterial Vessel Area, Venous Vessel Area, and Vascular Area within 1.0–1.5 PD) were collectively incorporated into the model construction.

**TABLE 2:** Results of single-factor and multiple-factor regression on the training set.

Name	Desc	ΔAL ≤0.2 mm (N = 189)	ΔAL >0.2 mm (N = 366)	OR (univariable)	OR (multivariable)
Sex	Male	113 (59.8%)	166 (45.4%)	​	​
​	Female	76 (40.2%)	200 (54.6%)	1.79 (1.25-2.56, p = 0.001)	3.06 (1.29-7.24, **p = 0.011**)
Presence of leopard spot lesions	Existence	144 (76.2%)	140 (38.3%)	​	​
​	Non-existent	45 (23.8%)	226 (61.7%)	5.17 (3.48-7.67, p < 0.001)	10.95 (4.81-24.92, **p < 0.001**)
DS	Mean ± SD	0.8 ± 1.1	0.4 ± 1.1	0.67 (0.56-0.81, p < 0.001)	0.37 (0.11-1.25, p = 0.110)
AL	Mean ± SD	23.1 ± 0.8	23.2 ± 0.8	1.25 (1.00-1.56, p = 0.049)	0.82 (0.44-1.50, p = 0.516)
SE	Mean ± SD	0.4 ± 1.2	0.1 ± 1.1	0.74 (0.62-0.88, p < 0.001)	2.31 (0.74-7.24, p = 0.149)
Vascular fractal dimension	Mean ± SD	1.6 ± 0.0	1.5 ± 0.0	0.00 (0.00-0.00, p < 0.001)	0.00 (0.00-0.00, **p < 0.001**)
Vascular fractal dimension in the inferior disk region	Mean ± SD	1.4 ± 0.0	1.3 ± 0.0	0.00 (0.00-0.00, p < 0.001)	23.18 (0.00-140609113.06, p = 0.693)
Vascular density	Mean ± SD	0.1 ± 0.0	0.1 ± 0.0	0.00 (0.00-0.00, p < 0.001)	0.00 (0.00-2595892755.06, p = 0.352)
Vascular coverage density between 0.5-1.0 PD	Mean ± SD	0.1 ± 0.0	0.1 ± 0.0	0.00 (0.00-0.00, p < 0.001)	0.00 (0.00-0.00, **p < 0.001**)
Vascular coverage density between 2.0-2.5 PD	Mean ± SD	0.1 ± 0.0	0.1 ± 0.0	0.00 (0.00-0.63, p = 0.043)	4758372422945087579110082653192192.00 (3205269722583626.00-7064025830934859015488922918641682270052866511077376.00, **p < 0.001**)
Average vascular density in the inferior region	Mean ± SD	0.1 ± 0.0	0.1 ± 0.0	0.00 (0.00-0.00, p < 0.001)	0.00 (0.00-0.00, **p = 0.002**)
Venous centerline fractal dimension	Mean ± SD	1.3 ± 0.0	1.3 ± 0.0	0.01 (0.00-1.00, p = 0.050)	245108861388.53 (0.02-3186040700930394712702976.00, p = 0.089)
Venous fractal dimension	Mean ± SD	1.5 ± 0.0	1.4 ± 0.1	0.00 (0.00-0.00, p < 0.001)	0.00 (0.00-0.00, **p < 0.001**)
Venous vessel area	Mean ± SD	6.2 ± 0.8	5.7 ± 0.8	0.36 (0.27-0.47, p < 0.001)	14.83 (2.19-100.37, **p = 0.006**)
Vascular area	Mean ± SD	10.6 ± 1.1	9.7 ± 1.3	0.54 (0.46-0.65, p < 0.001)	1.72 (0.79-3.75, p = 0.176)
Vascular area within 0.5–1.0 PD	Mean ± SD	1.4 ± 0.2	1.4 ± 0.2	0.47 (0.22-0.99, p = 0.048)	0.66 (0.04-10.06, p = 0.768)
Vascular area within 1.0–1.5 PD	Mean ± SD	1.7 ± 0.3	1.6 ± 0.3	0.55 (0.31-0.98, p = 0.044)	34.87 (3.79-320.42, **p = 0.002**)
Vascular area within 2.0–2.5 PD	Mean ± SD	1.5 ± 0.2	1.3 ± 0.2	0.01 (0.00-0.03, p < 0.001)	0.00 (0.00-0.01, **p < 0.001**)

Bold values indicate P < 0.05, which is statistically significant.

Hyperparameter tuning using fivefold cross-validation yielded the optimal parameter sets for all candidate algorithms (Table 3). The decision tree performed best with a maximum depth of 5 and a minimum split size of 10. The RF model achieved its best performance with 150 estimators and two features per split. For gradient boosting methods, XGBoost performed optimally with a learning rate of 0.1, 100 estimators, and a maximum depth of 3, whereas LightGBM required 100 estimators, 31 leaves, and subsampling and column-sampling rates of 0.6. The SVM model showed optimal performance with a linear kernel and C = 0.1, and the ANN model achieved the best results with two hidden layers of 50 units and a tanh activation function.

**TABLE 3:** Optimal parameters of each model on the training set.

Model	Optimal parameters
Decision tree	‘Ccp alpha’: 0.01, ‘max depth’: 5, ‘max features’: None, ‘min samples split’: 10
Random forest	N estimators = 150, max features = 2
XGBoost	‘Learning rate’: 0.1, ‘max depth’: 3, ‘n estimators’: 100, ‘subsample’: 1.0
LightGBM	‘Colsample bytree’: 0.6, ‘learning rate’: 0.1, ‘n estimators’: 100, ‘num leaves’: 31, ‘subsample’: 0.6
SVM	‘C': 0.1, ‘degree’: 2, ‘gamma’: ‘Scale’, ‘kernel’: ‘Linear’
ANN	‘Activation’: ‘Tanh’, ‘hidden layer sizes’: (50, 50)

Model performance metrics for the validation cohort are summarized in Table 4. The AUC values ranged from 0.852 (SVM) to 0.965 (LightGBM). In addition to AUC, accuracy, precision, sensitivity, specificity, F1 score, Cohen’s kappa, and Youden’s index, these metrics demonstrated variable performance across algorithms. The RF model showed a high AUC (0.961; 95% CI, 0.933–0.984) and the highest F1 score among all evaluated models; therefore, it was selected as the final prediction model.

**TABLE 4:** Performance comparison of models on the validation set.

Model	AUC	95% CI lower	95% CI upper	Accuracy	Precision	Sensitivity	Specificity	F1 score	Kappa	Youden’s J
Logistic	0.96	0.93	0.98	0.90	0.95	0.89	0.91	0.92	0.78	0.81
Decision tree	0.95	0.92	0.98	0.89	0.92	0.91	0.85	0.92	0.76	0.76
Random forest	0.96	0.93	0.98	0.89	0.92	0.91	0.85	0.92	0.76	0.76
XGBoost	0.96	0.93	0.98	0.89	0.93	0.90	0.87	0.92	0.76	0.77
LightGBM	0.97	0.94	0.99	0.91	0.93	0.92	0.87	0.93	0.79	0.80
SVM	0.85	0.78	0.91	0.75	0.76	0.91	0.45	0.83	0.40	0.36
ANN	0.92	0.88	0.96	0.83	0.86	0.89	0.72	0.88	0.62	0.61

**FIGURE 3:**
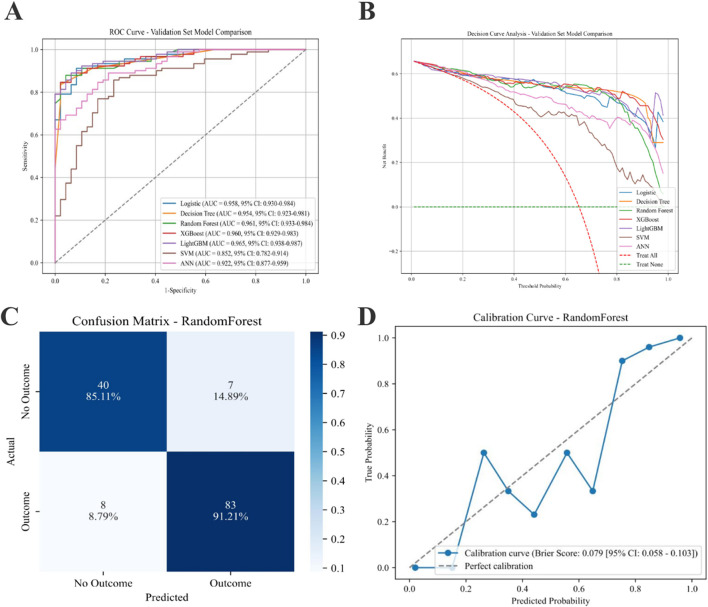
Performance of various predictive models on the AL progression validation set. **(A)** ROC curves for the ML models. **(B)** Seven models of the Decision Curve Analysis for the Validation Sets. **(C)** Confusion matrix for the best model (RF). **(D)** Calibration Curve for the best model (RF). AL: Axial Length; ML: machine learning; RF: Random Forest.

Figure 3 presents the comparative performance of all models. The ROC curves for the seven algorithms are displayed in Figure 3A. Decision curve analysis for the validation set is shown in Figure 3B. The RF Model exhibits superior performance. The confusion matrix for the RF Model (Figure 3C) depicts classification outcomes, and the calibration curve (Figure 3D) shows the agreement between predicted and observed probabilities.

**FIGURE 4:**
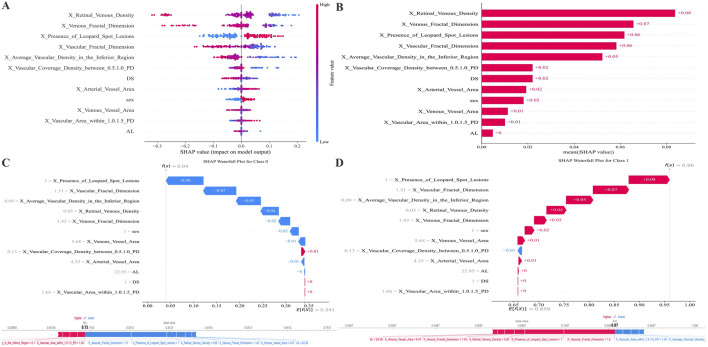
SHAP-based feature attribution for the RF model. **(A)** Mean absolute SHAP values rank feature importance. **(B)** SHAP summary plot illustrating the direction and magnitude of each feature’s effect (red: high value, blue: low value). **(C,D)** SHAP waterfall plots for representative Class 0 (AL progression <0.2 mm) and Class 1 (AL progression ≥0.2 mm) predictions, showing features driving risk upward (red) or downward (blue). The figure below attempts to plot the features from the previous figure along a straight line.

Feature attribution for the final RF model was assessed using SHAP (Figure 4). The mean absolute SHAP value plot (Figure 4A) displays the ranked contribution of each variable to the model output, with higher values indicating greater influence on the prediction. The SHAP summary plot (Figure 4B) presents the distribution of SHAP values for all features across the dataset, showing the range and dispersion of each feature’s effect on the predicted probability of AL progression. The color gradient reflects the relative magnitude of the underlying feature values. SHAP analysis indicates that the more essential features in the predictive model are the Retinal Venous Density, Venous Fractal Dimension, Presence of Leopard Spot Lesions, Vascular Fractal Dimension, and Average Vascular Density in the Inferior Region.

Instance-level interpretability is demonstrated in the waterfall plots (Figures 4C,D). For a representative case classified as no progression (AL ≤ 0.2 mm), Figure 4C shows how individual features cumulatively shift the model output toward a lower predicted risk. For a representative case classified as progression (AL > 0.2 mm), Figure 4D depicts the sequential contribution of features that increase the expected probability of progression. The supplemental linear arrangement of SHAP values provides an aligned visualization of feature effects for comparison across samples.

**FIGURE 5:**
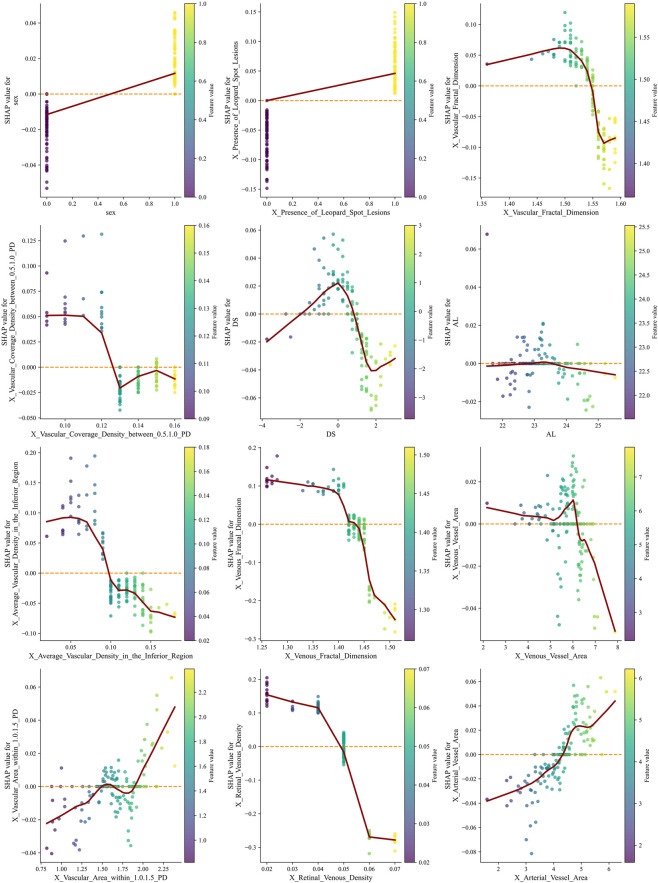
SHAP summary plots illustrate the contribution of demographic, structural, and retinal microvascular features to the RF model predicting AL progression. Each point represents one individual eye. The horizontal axis represents the SHAP value, indicating the marginal contribution of each feature to the predicted probability of AL progression (greater than 0.2 mm). Positive SHAP values indicate an increased predicted risk of axial elongation, whereas negative values indicate a protective or risk-reducing effect. Points are color-coded according to the normalized feature value, with red representing higher feature values and blue representing lower feature values. The vertical dispersion of points for each variable reflects inter-individual variability and potential nonlinear effects that the model captures. Lower retinal venous density and reduced venous or overall vascular fractal dimensions are associated with predominantly positive SHAP values at lower feature levels, indicating that microvascular rarefaction markedly increases the predicted risk of AL progression. In contrast, higher vascular coverage density and increased vascular area within specific annular regions (0.5–1.0 PD and 1.0–1.5 PD from the optic disc) show positive SHAP contributions at higher feature values, suggesting a region-specific compensatory vascular response. The presence of leopard-spot lesions consistently demonstrates positive SHAP values across observations, highlighting a strong association with accelerated axial elongation. Sex and baseline AL exhibit relatively smaller but discernible contributions, indicating their supportive role within the multivariable prediction framework rather than dominant driving factors. AL, axial length; PD, papillary diameter; SHAP, Shapley Additive Explanations; RF, Random Forest.

SHAP summary plots were generated to visualize the contribution of each feature to the RF model’s prediction of AL elongation. As shown in Figure 5, multiple demographic, structural, and retinal microvascular parameters significantly influenced the predicted probability of AL progression. The presence of leopard-spot lesions showed consistently positive SHAP values across observations. Vascular and venous fractal dimensions showed a broad distribution of SHAP values, with lower feature levels generally associated with higher positive contributions. Several regional vascular metrics, including vascular coverage density between 0.5 and 1.0 PD, average vascular density in the inferior region, venous vessel area, and vascular area within 1.0–1.5 PD, also displayed positive SHAP contributions at varying feature levels. Retinal venous density showed a wide range of SHAP values, with lower values corresponding to higher positive contributions in the model. Additional variables such as base AL, arterial vessel area, and sex demonstrated relatively more minor but discernible contributions.

## Discussion

To balance statistical significance and clinical relevance, we integrated statistical variable screening with clinical expert judgment, selecting sex, DS, AL, and nine quantitative retinal parameters to enhance the model’s interpretability and clinical acceptability. The RF model was ultimately chosen as the optimal model due to its balanced performance, characterized by high AUC values, accuracy, recall, and F1 score. This model achieves a good balance between accurately identifying progressive cases and avoiding false negatives by identifying retinal microvascular features (particularly venous-related parameters and fractal dimension) and leopard spot lesions as key predictors of AL progression. These findings provide new insights and potential biomarkers to understand the mechanisms of AL progression in children and to develop effective intervention strategies. To our knowledge, this study represents an early attempt to construct a predictive model for short-term AL progression in children using quantitatively derived CFP features. In particular, the inclusion of venous-related microvascular parameters, such as venous density and venous fractal dimension, extends existing CFP-based research beyond traditional structural descriptors.

This study analyzed basic information, including sex, DS, and baseline AL. The results showed that sex was one of the factors related to myopia progression, which was consistent with previous studies. Lee SS et al. found that female participants exhibited faster myopia progression and greater AL elongation (Lee et al., 2022). Mingming C et al. also found that the myopia rate in females was significantly higher than in males (Mingming et al., 2025). Many scholars also include sex in the prediction model of myopia progression and the myopia prevention and control effect (Yii et al., 2025; Gopalakrishnan et al., 2024; Fan et al., 2022; Xu et al., 2025). Our study found that female sex was also a factor in the myopia progression of school-age children. Surprisingly, DS and baseline AL showed limited significance in logistic regression analysis. This may be attributed to the study’s screening-based data, which included children with hyperopia reserve or mild-to-moderate myopia, with a narrow age range (6–10 years). Consequently, these parameters had limited predictive value for AL progression, underscoring the critical importance of dynamic AL monitoring. Although sex differences were incorporated into the prediction model, sex-stratified analyses of baseline refractive and biometric parameters were not the primary focus of this study and warrant further investigation in dedicated longitudinal analyses.

In the final model, the importance of microvascular parameters such as retinal venous density, venous fractal dimension, and vascular fractal dimension even surpasses that of some traditional refractive biomarkers (such as baseline AL and SE). This finding further validates the conclusion by Zhao M et al. that morphological parameters of vascular microstructure could serve as a potential biomarker for monitoring the progression of AL (Zhao et al., 2022). Wang Q et al. found that the choroidal Sattler’s and Haller’s layer densities below the macular fovea are critical for monitoring changes in myopia progression (Wang Q. et al., 2025). This is like the conclusion that the average vascular density below the macula is significant in this study. In previous studies, our team also included vascular density as a relevant factor for high myopia in Chinese college students (Zhang et al., 2023). Many scholars have also shown that vascular density is an independent risk factor and an early predictor of myopia progression (Veselinović et al., 2025; Cheng et al., 2019). Fractal dimension serves as a quantitative measure of vascular network complexity, with its reduction typically indicating structural simplification or sparsification. Our study demonstrates that lower vascular and venous fractal dimensions are associated with an elevated risk of AL progression. Previous research has also reported that decreased vascular fractal dimension is strongly associated with reduced choroidal and retinal perfusion, which may constitute a critical pathological basis for sustained AL growth and fundus alterations (Furnon et al., 2023). This phenomenon indicates that during rapid AL elongation, the choroid and retina undergo abnormal or adaptive changes in blood supply, leading to remodeling of the microvascular network (Zhu et al., 2025; Chen et al., 2025). Studies also suggest that myopia acts as a protective factor against diabetic retinopathy (Wang et al., 2016; Fu et al., 2016), likely due to its reduced vascular fractal dimension. The simplified vascular architecture may partially preserve the morphological integrity of microvessels. Meanwhile, the leopard spot lesion, as an essential structural feature, was identified as a strong predictor, possibly related to choroidal thinning and scleral remodeling. The association between choroidal thickness and choroidal vascular density mediated by AL further supports the central role of posterior pole structural changes in myopia progression (Yang et al., 2025).

Interpretation of the SHAP values from the RF model revealed a coherent vascular mechanism underlying AL elongation. Recent research by Wang et al., which quantified CFP for the interpretable analysis of HM in adults, identified particular optic disc and atrophic areas, along with vascular diameter, as effective predictors (Zou et al., 2025). Our study focuses on early changes in childhood myopia and has discovered biomarkers associated with AL progression in children. Lower retinal venous density and reduced venous or overall vascular fractal dimensions were associated with strong positive SHAP values, indicating that microvascular rarefaction markedly increased the probability of AL progression. Such attenuation of the retinal venous network likely reflects early perfusion insufficiency and localized metabolic stress. These hemodynamic alterations may trigger remodeling of the choroid–sclera complex, reduce scleral stiffness, and predispose the eye to accelerated AL elongation. In contrast, higher vascular coverage or vascular area within specific perifoveal and mid-peripheral regions (e.g., 0.5–1.0 PD and 1.0–1.5 PD zones) showed positive SHAP contributions at higher feature values, suggesting a secondary, compensatory dilation response to hypoxia. Although such dilation may occur to enhance perfusion, it likely represents maladaptive vascular remodeling that further destabilizes ocular wall biomechanics. The substantial positive contribution of leopard-spot lesions further supports this interpretation, marking regions of chronic chorioretinal stress and structural vulnerability. Collectively, the model suggests that global venous rarefaction, combined with region-specific compensatory vascular dilation, creates an imbalance in retinal and scleral biomechanics that increases the likelihood of AL elongation. Based on the ranking of variable importance within the predictive model used in this study, we hypothesize that the density and complexity of early-stage vascular development in childhood AL progression may precede the emergence of leopard spots or contribute to their appearance. However, because leopard spot density was suboptimally quantified in early childhood, our study employed a binary classification (present or absent). This categorical variable may also alter the ranking of variable importance.

This study has the following limitations: First, due to the lack of external validation, the results may be influenced by specific equipment, raising questions about the model’s generalization ability. Future research should expand the sample size for external validation. Second, all CFP images were acquired using a single imaging device in one city, which may limit the generalizability of the findings to other populations or imaging systems. Hybrid models integrating raw CFP images and quantitative imageomics features may further enhance predictive performance while preserving interpretability. Additionally, potential unrecorded variations in routine refractive correction during follow-up may have influenced AL progression. Finally, although relative CFP-derived metrics were used to reduce AL–related magnification effects, no formal AL–based image rescaling was applied. Future studies incorporating Littmann or Bennett correction models may further improve measurement accuracy, particularly in populations with extreme AL elongation.

## Conclusion

In this school-based cohort of children aged 6–10 years, a machine learning model incorporating quantitative CFP parameters and clinical characteristics demonstrated high accuracy in predicting 1-year AL progression. Retinal microvascular complexity, vessel density metrics, venous structural features, and the presence of leopard-spot lesions emerged as key contributors to model performance. The RF model showed superior discrimination and calibration compared with other algorithms. These findings indicate that CFP-derived biomarkers provide valuable information for identifying children at elevated risk for rapid axial elongation and may support earlier, individualized myopia control interventions. Future studies focusing on causal inference and growth trajectory modeling may further clarify the interaction between age, baseline AL, refractive error, and axial elongation rate.

## Data Availability

The raw data supporting the conclusions of this article will be made available by the authors, without undue reservation.
