# Supplementary material for: Quantitative color fundus photography parameters as potential biomarkers of axial length progression: evidence from a machine learning cohort study
Source: Front Cell Dev Biol. 2026 Jan 26;14:1753213. doi: 10.3389/fcell.2026.1753213 (PMC12883787; doi:10.3389/fcell.2026.1753213)
Supplement: Supplementary file 1 [file DataSheet1.docx]

**S1.1 Quantitative CFP Segmentation Method and Model Validation**

EVisionAI Comprehensive Fundus Examination System (Version 2.0), developed by EVision Technology (Beijing) Co., Ltd. The deep learning models utilized in this study were not custom-trained de novo for this specific project; rather, they were pre-trained on large, multi-center datasets and then applied to the study images for inference. This approach leverages a robust, standardized model that has undergone extensive external validation.

The analytical pipeline consists of several key stages, each validated for reliability: **(1) Pre-processing:** All color fundus photographs underwent a standardized pre-processing pipeline to minimize variability. This included: automated Region of Interest (ROI) extraction using multi-channel separation and red-channel thresholding; denoising via Gaussian low-pass filtering; normalization of brightness and color in the LAB color space to standardize appearance; and contrast enhancement using Contrast-Limited Adaptive Histogram Equalization (CLAHE) to improve vascular visibility [Shao et al., 2021]. **(2) Segmentation:** The core segmentation tasks were performed using dedicated deep learning architectures. Retinal Vessel Segmentation: A ResNet101-UNet semantic segmentation model was employed. This architecture combines the powerful feature extraction capabilities of a ResNet-101 encoder with the precise localization of a U-Net decoder, enabling accurate capture of multi-scale vascular features. Optic Disc and Cup Segmentation: A deep target detection network, integrated with edge extraction algorithms based on visual attention mechanisms, was used for precise optic nerve head structure identification [Zhang et al., 2022]. **(3) Derived Features (Vascular Parameters)**: Based on the segmentation results, quantitative parameters were automatically calculated. These included vascular fractal dimension (VDf), vascular caliber (VC), vascular tortuosity (VT), vascular density (VD), vascular branching angle (VBA), arteriole-to-venule ratio (AVR), optic disc area, and cup-to-disc ratio (C/D). Vessel centerlines were extracted via bidirectional morphological erosion, and boundaries were refined to sub-pixel precision using the Canny edge detector. Arteries and veins were distinguished based on a combination of color intensity, luminance distribution, and topological features. **(4)Grader Details and Model Training (Reference Standard):** The reference standard for training the vessel segmentation model was established through a rigorous, machine-assisted semi-automatic annotation process. Initial vessel extraction was performed algorithmically, followed by independent correction, verification, and refinement by two trained human graders. The inter-observer agreement for these manual annotations was excellent, with a kappa statistic (κ) exceeding 0.80 [Huang et al., 2023], ensuring a high-quality ground truth dataset. **(5)Validation of Intermediate Steps:** The performance of the segmentation models was rigorously evaluated against the expert-annotated ground truth. For the retinal vessel segmentation task, the model achieved a high Dice similarity coefficient of 0.9766, indicating excellent pixel-wise agreement with human graders [He et al., 2023]. Furthermore, the quantitative measurements of derived parameters (e.g., VC, VD) demonstrated high reliability. The inter-class correlation coefficient (ICC) for key vascular parameters, assessed across repeated measurements, consistently exceeded 0.90, indicating excellent measurement reproducibility. **(6)Pixel Calibration for Quantitative Accuracy:** To ensure spatial measurements were consistent across different camera models, a camera-agnostic pixel calibration method was applied. This method leverages the automated detection of the ROI and optic disc to estimate the pixel pitch, achieving an error of less than 5% compared to ISO-standard manual measurements [Long et al., 2022]. This step is critical for the accurate, device-independent quantification of morphometric parameters like vascular caliber and disc area.

1. He HL, Liu YX, Chen XY, et al. Fundus Tessellated Density of Pathologic Myopia. Asia Pac J Ophthalmol (Phila). 2023;12(6):604-613.
2. Huang D, Qian Y, Yan Q, et al. Prevalence of Fundus Tessellation and Its Screening Based on Artificial Intelligence in Chinese Children: the Nanjing Eye Study. Ophthalmol Ther. 2023;12(5):2671-2685.
3. Long T, Xu Y, Zou H, et al. A Generic Pixel Pitch Calibration Method for Fundus Camera via Automated ROI Extraction. Sensors (Basel). 2022;22(21):8565.
4. Shao L, Zhang QL, Long TF, et al. Quantitative Assessment of Fundus Tessellated Density and Associated Factors in Fundus Images Using Artificial Intelligence. Transl Vis Sci Technol. 2021;10(9):23.
5. Zhang G, et al. Multi-Model Domain Adaptation for Diabetic Retinopathy Classification. Front Physiol. 2022;13:918929.

**S1.2 Visualization of Key Quantitative Metrics [1,2]**


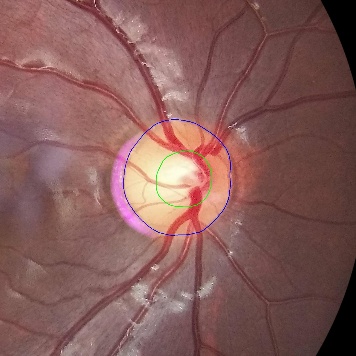


| Optic cup segmentation |  |
| --- | --- |


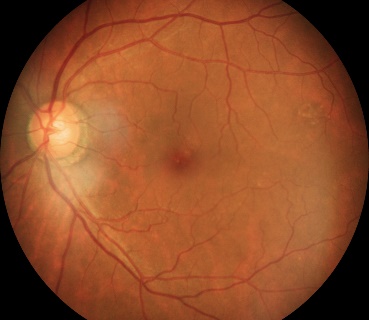

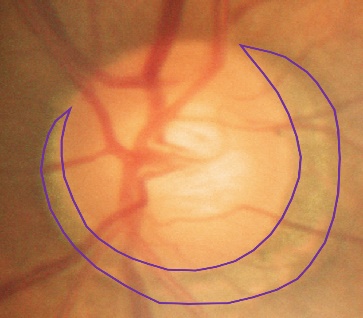

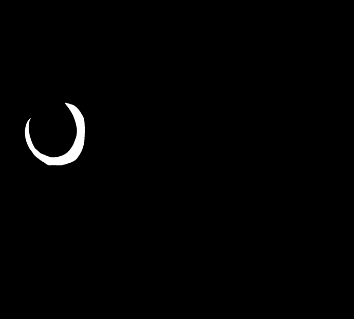


S2. Quantified CFP Variable Table and Variable Types

Parapapillary atrophy

Segmentation of Optic Disc Atrophy


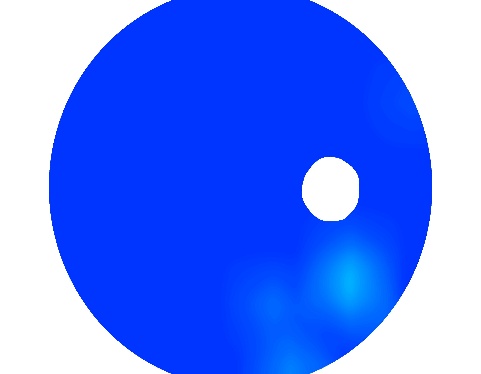


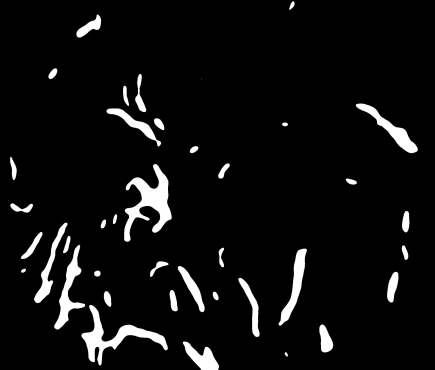

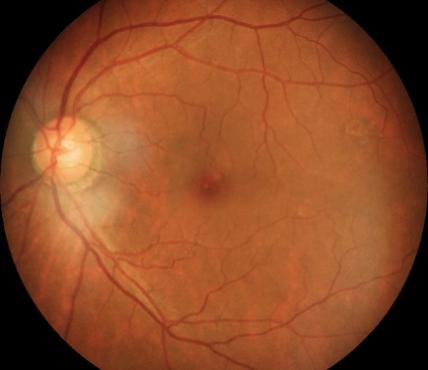


fundus tessellation segmentation


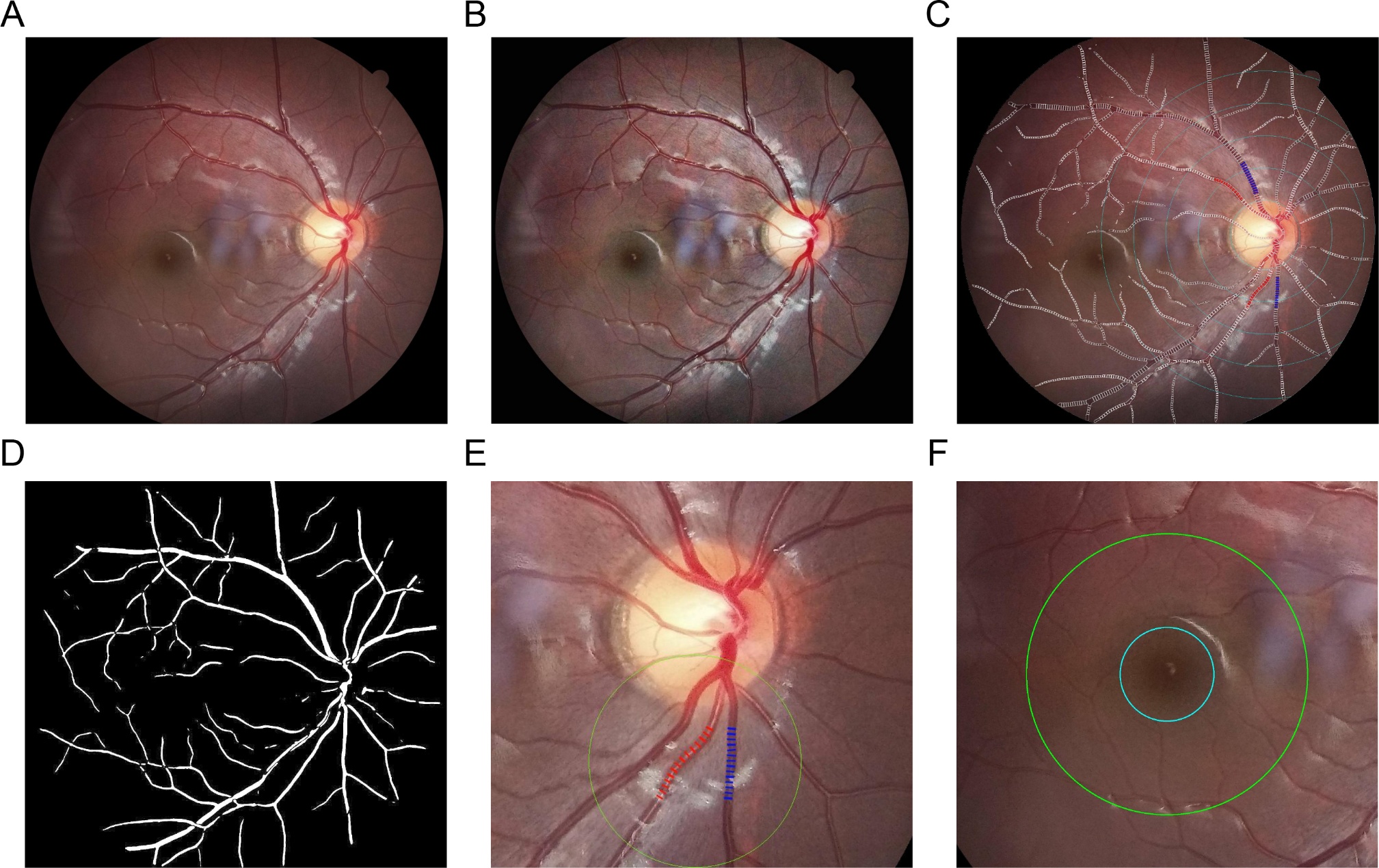


This schematic illustrates the identification (C), segmentation (D), arterial and venous local details (E), and macular detail display (F) of blood vessels after image enhancement processing (B).

[1] Zou H, Liu J, Shi S, et al. Retinal features as predictive indicators for high myopia: insights from explainable multi-machine learning models. Front Bioeng Biotechnol. 2025;13:1609639.

[2] Zhang X, Wang Z, Yu J, Wang J, Song D, Zhang B, Li X, Du B, Wei R. Predicting myopia risk using a machine learning model based on fundus imageomics. Sci Rep. 2025 Dec 12;15(1):43729.

**S1.3 Quantified CFP Variable Table and Variable Types**

| **variables** | **Variable Type** |
| --- | --- |
| Presence of atrophic regions | Categorical variable |
| Presence of leopard spots | Categorical variable |
| Average vessel branch angle | Continuous variable |
| Vessel length (μm) | Continuous variable |
| Artery (centerline) linear fractal dimension | Continuous variable |
| Vessel (centerline) linear fractal dimension | Continuous variable |
| Vein (centerline) linear fractal dimension | Continuous variable |
| Vessel fractal dimension | Continuous variable |
| Vessel fractal dimension within inferior optic disc region | Continuous variable |
| Vessel fractal dimension within nasal optic disc region | Continuous variable |
| Vessel fractal dimension within superior optic disc region | Continuous variable |
| Vessel fractal dimension within temporal optic disc region | Continuous variable |
| Average vessel diameter (μm) | Continuous variable |
| Average vessel diameter within 0.5–1.0 PD | Continuous variable |
| Average vessel diameter within 1.0–1.5 PD | Continuous variable |
| Average vessel diameter within 1.5–2.0 PD | Continuous variable |
| Average vessel diameter within 2.0–2.5 PD | Continuous variable |
| Average vessel diameter within 3 mm central foveal region | Continuous variable |
| Average vessel diameter within 5 mm central foveal region | Continuous variable |
| Average vessel diameter within superior optic disc region | Continuous variable |
| Average vessel diameter within temporal optic disc region | Continuous variable |
| Average vessel diameter within inferior optic disc region | Continuous variable |
| Average vessel diameter within nasal optic disc region | Continuous variable |
| Average vessel curvature | Continuous variable |
| Average vessel curvature within 0.5–1.0 PD | Continuous variable |
| Average vessel curvature within 1.0–1.5 PD | Continuous variable |
| Average vessel curvature within 1.5–2.0 PD | Continuous variable |
| Average vessel curvature within 2.0–2.5 PD | Continuous variable |
| Average arterial curvature within 0.5–1.0 PD | Continuous variable |
| Average arterial curvature within 1.0–1.5 PD | Continuous variable |
| Average arterial curvature within 1.5–2.0 PD | Continuous variable |
| Average arterial curvature within 2.0–2.5 PD | Continuous variable |
| Average venous curvature within 0.5–1.0 PD | Continuous variable |
| Average venous curvature within 1.0–1.5 PD | Continuous variable |
| Average venous curvature within 1.5–2.0 PD | Continuous variable |
| Average venous curvature within 2.0–2.5 PD | Continuous variable |
| Vessel tortuosity within superior optic disc region | Continuous variable |
| Vessel tortuosity within temporal optic disc region | Continuous variable |
| Vessel tortuosity within inferior optic disc region | Continuous variable |
| Vessel tortuosity within nasal optic disc region | Continuous variable |
| Average arterial tortuosity | Continuous variable |
| Arterial tortuosity within superior optic disc region | Continuous variable |
| Arterial tortuosity within temporal optic disc region | Continuous variable |
| Arterial tortuosity within inferior optic disc region | Continuous variable |
| Arterial tortuosity within nasal optic disc region | Continuous variable |
| Average venous tortuosity | Continuous variable |
| Vessel tortuosity within 3 mm central foveal region | Continuous variable |
| Vessel tortuosity within 5 mm central foveal region | Continuous variable |
| Arterial tortuosity within 3 mm central foveal region | Continuous variable |
| Arterial tortuosity within 5 mm central foveal region | Continuous variable |
| Venous tortuosity within 3 mm central foveal region | Continuous variable |
| Venous tortuosity within 5 mm central foveal region | Continuous variable |
| Venous tortuosity within superior optic disc region | Continuous variable |
| Venous tortuosity within temporal optic disc region | Continuous variable |
| Venous tortuosity within inferior optic disc region | Continuous variable |
| Venous tortuosity within nasal optic disc region | Continuous variable |
| Vessel density | Continuous variable |
| Vessel coverage density within 0.5–1.0 PD | Continuous variable |
| Vessel coverage density within 1.0–1.5 PD | Continuous variable |
| Vessel coverage density within 1.5–2.0 PD | Continuous variable |
| Vessel coverage density within 2.0–2.5 PD | Continuous variable |
| Average vessel density within 3 mm central foveal region | Continuous variable |
| Average vessel density within 5 mm central foveal region | Continuous variable |
| Average vessel density within superior region | Continuous variable |
| Average vessel density within temporal region | Continuous variable |
| Average vessel density within inferior region | Continuous variable |
| Average vessel density within nasal region | Continuous variable |
| Average arterial diameter (μm) | Continuous variable |
| Average arterial diameter within 0.5–1.0 PD | Continuous variable |
| Average arterial diameter within 1.0–1.5 PD | Continuous variable |
| Average arterial diameter within 1.5–2.0 PD | Continuous variable |
| Average arterial diameter within 2.0–2.5 PD | Continuous variable |
| Arterial diameter within 3 mm central foveal region | Continuous variable |
| Arterial diameter within 5 mm central foveal region | Continuous variable |
| Arterial diameter within superior optic disc region | Continuous variable |
| Arterial diameter within temporal optic disc region | Continuous variable |
| Arterial diameter within inferior optic disc region | Continuous variable |
| Arterial diameter within nasal optic disc region | Continuous variable |
| Average venous diameter (μm) | Continuous variable |
| Average venous diameter within 0.5–1.0 PD | Continuous variable |
| Average venous diameter within 1.0–1.5 PD | Continuous variable |
| Average venous diameter within 1.5–2.0 PD | Continuous variable |
| Average venous diameter within 2.0–2.5 PD | Continuous variable |
| Venous diameter within 3 mm central foveal region | Continuous variable |
| Venous diameter within 5 mm central foveal region | Continuous variable |
| Venous diameter within superior optic disc region | Continuous variable |
| Venous diameter within temporal optic disc region | Continuous variable |
| Venous diameter within inferior optic disc region | Continuous variable |
| Venous diameter within nasal optic disc region | Continuous variable |
| Arteriovenous diameter ratio (AVR) | Continuous variable |
| Angle between vascular arch and macula (degrees) | Continuous variable |
| Distance between vascular arch and macula intersection (μm) | Continuous variable |
| Angle between vascular arch and 1 PD outside optic disc (degrees) | Continuous variable |
| Distance between vascular arch and 1 PD outside optic disc (μm) | Continuous variable |
| Angle between vascular arch and 2 PD outside optic disc (degrees) | Continuous variable |
| Distance between vascular arch and 2 PD outside optic disc (μm) | Continuous variable |
| Cup area (mm²) | Continuous variable |
| Cup horizontal diameter (μm) | Continuous variable |
| Cup vertical diameter (μm) | Continuous variable |
| Cup circularity | Continuous variable |
| Cup-to-disc area ratio (CDAR) | Continuous variable |
| Horizontal cup-to-disc ratio (CDR-H) | Continuous variable |
| Vertical cup-to-disc ratio (CDR-V) | Continuous variable |
| Rim I distance (μm) | Continuous variable |
| Rim N distance (μm) | Continuous variable |
| Rim S distance (μm) | Continuous variable |
| Rim T distance (μm) | Continuous variable |
| Optic disc vertical-to-horizontal ratio (vessel arch axis) | Continuous variable |
| Optic disc short-to-long axis ratio | Continuous variable |
| Angle between optic disc long axis and vertical line | Continuous variable |
| Maximum disc–cup rim distance | Continuous variable |
| Minimum disc–cup rim distance | Continuous variable |
| Angle of minimum disc–cup rim distance relative to horizontal (start) | Continuous variable |
| Angle of minimum disc–cup rim distance relative to horizontal (end) | Continuous variable |
| Disc area (mm²) | Continuous variable |
| Disc horizontal diameter (μm) | Continuous variable |
| Disc vertical diameter (μm) | Continuous variable |
| Disc circularity | Continuous variable |
| Optic disc tilt angle (degrees) | Continuous variable |
| Distance from optic disc center to boundary (μm) | Continuous variable |
| Distance from optic disc to macula (μm) | Continuous variable |
| Angle between macula–disc line and reference (degrees) | Continuous variable |
| Optic disc long-to-short axis ratio | Continuous variable |
| Retinal arterial density | Continuous variable |
| Retinal venous density | Continuous variable |
| Arterial fractal dimension | Continuous variable |
| Venous fractal dimension | Continuous variable |
| Arterial vessel length | Continuous variable |
| Venous vessel length | Continuous variable |
| Main vascular arch arteriovenous ratio | Continuous variable |
| Inferior main vascular arch arteriovenous ratio | Continuous variable |
| Superior main vascular arch arteriovenous ratio | Continuous variable |
| Average arterial branch angle | Continuous variable |
| Average venous branch angle | Continuous variable |
| Distance from foveal center to boundary (μm) | Continuous variable |
| Angle between disc–macula line and optic disc long axis | Continuous variable |
| Distance from nasal disc margin to foveal center (μm) | Continuous variable |
| Arterial vessel area | Continuous variable |
| Venous vessel area | Continuous variable |
| Total vessel area | Continuous variable |
| Vessel area within 0.5–1.0 PD | Continuous variable |
| Vessel area within 1.0–1.5 PD | Continuous variable |
| Vessel area within 1.5–2.0 PD | Continuous variable |
| Vessel area within 2.0–2.5 PD | Continuous variable |

**S1.4 Definition of Quantitative Parameters [1]**

| Optic disc area | The area of the optic disc obtained by segmentation |
| --- | --- |
| Horizontal diameter of the optic disc | The diameter in the horizontal direction of the minimal circumscribed circle of the optic disc |
| Vertical diameter of the optic disc | The diameter in the vertical direction of the minimal circumscribed circle of the optic disc |
| Optic cup area | The area of the optic cup obtained by segmentation |
| Horizontal diameter of the optic cup | The diameter in the horizontal direction of the minimal circumscribed circle of the optic cup |
| Vertical diameter of the optic cup | The diameter in the vertical direction of the minimal circumscribed circle of the optic cup |
| Cup-to-disc area ratio | The area ratio of the optic cup to the optic disc |
| Horizontal cup-to-disc ratio | Ratio of the horizontal diameter of the optic cup to the horizontal diameter of the optic disc |
| Vertical cup-to-disc ratio | The ratio of the vertical diameter of the optic cup to the vertical diameter of the optic disc |
| Parapapillary atrophy area | The area of the Parapapillary atrophy |
| Height of parapapillary atrophy | The length of the minimal external matrix in the vertical direction of the parapapillary atrophy |
| Width of parapapillary atrophy | The distance between the point on the central line of the parapapillary atrophy and the shortest segment of the intersection of the two arc-shaped edges |
| Parapapillary atrophy-to-optical disc area ratio | The area ratio of the parapapillary atrophy to the optic disc |
| Width of parapapillary atrophy-to-horizontal diameter of the optic disc ratio | The ratio of the width of the parapapillary atrophy to the horizontal diameter of the optic disc |
| Height of parapapillary atrophy-to-vertical diameter of the optic disc ratio | The ratio of the height of the parapapillary atrophy to the vertical diameter of the optic disc |
| Width of inferior rim | Distance between the boundary of the optic disc and the optic cup on the inferior side in the vertical direction of the optic disc |
| Width of superior rim | Distance between the upper side of the optic disc and the optic cup boundary in the vertical direction of the optic disc |
| Width of nasal rim | Distance between the nasal side of the optic disc and the optic cup boundary in the horizontal direction of the optic disc |
| Width of temporal rim | Distance between the temporal disc and cup boundaries in the horizontal direction of the optic disc |
| Fractal dimension | A measure of vascular irregularity that reflects the complexity of retinal vascular morphology. The metric is calculated based on the counting box dimension method |
| Vessel density | The area of retinal blood vessels per unit area. |
| Vascular tortuosity | The mean of the curvature of the points on the center line of all vessels |
| Arterial tortuosity | The mean of the curvature of the points on the center line of all arteries |
| Venous tortuosity | The mean of the curvature of the points on the center line of all veins |
| Mean vessel diameter | The mean vessel diameter corresponding to the points on the center line of all vessels |
| Mean arterial diameter | The mean vessel diameter corresponding to the points on the center line of all arteries |
| Mean venous diameter | The mean vessel diameter corresponding to the points on the center line of all veins |
| Arterial-to-venous ratio | The ratio of the mean arterial vessel diameter to the mean venous vessel diameter |
| Tessellated density | The area of choroidal exposure per unit area |

[1] Zou H, Liu J, Shi S, et al. Retinal features as predictive indicators for high myopia: insights from explainable multi-machine learning models. Front Bioeng Biotechnol. 2025;13:1609639.

**S2. Baseline Information Form**

|  | level | Overall | 0 | 1 | p | test |
| --- | --- | --- | --- | --- | --- | --- |
| n |  | 693 | 236 | 457 |  |  |
| sex (%) | 0 | 345 (49.78) | 139 (58.90) | 206 (45.08) | 0.001 |  |
|  | 1 | 348 (50.22) | 97 (41.10) | 251 (54.92) |  |  |
| Presence of Atrophic Regions (%) | 0 | 489 (70.56) | 167 (70.76) | 322 (70.46) | 1 |  |
|  | 1 | 204 (29.44) | 69 (29.24) | 135 (29.54) |  |  |
| Presence of Leopard Spot Lesions (%) | 0 | 354 (51.08) | 182 (77.12) | 172 (37.64) | <0.001 |  |
|  | 1 | 339 (48.92) | 54 (22.88) | 285 (62.36) |  |  |
| Age (median [IQR]) |  | 8.00 [7.00, 8.00] | 8.00 [7.00, 9.00] | 8.00 [7.00, 8.00] | 0.448 | nonnorm |
| DS (median [IQR]) |  | 0.75 [0.00, 1.25] | 1.00 [0.50, 1.50] | 0.50 [0.00, 1.00] | <0.001 | nonnorm |
| AL (median [IQR]) |  | 23.15 [22.66, 23.70] | 23.08 [22.59, 23.62] | 23.21 [22.69, 23.75] | 0.043 | nonnorm |
| SE (median [IQR]) |  | 0.38 [-0.25, 1.00] | 0.75 [0.10, 1.25] | 0.25 [-0.38, 0.88] | <0.001 | nonnorm |
| Average Vascular Branching Angle (median [IQR]) |  | 64.15 [59.61, 68.04] | 65.10 [61.05, 68.77] | 63.86 [59.07, 67.86] | 0.048 | nonnorm |
| Vascular Fractal Dimension (median [IQR]) |  | 1.54 [1.52, 1.56] | 1.56 [1.54, 1.57] | 1.53 [1.51, 1.55] | <0.001 | nonnorm |
| Vascular Fractal Dimension in the Inferior Disk Region (median [IQR]) |  | 1.35 [1.33, 1.38] | 1.36 [1.34, 1.38] | 1.35 [1.32, 1.37] | <0.001 | nonnorm |
| Vascular Fractal Dimension in the Nasal Disk Region (median [IQR]) |  | 1.15 [1.07, 1.20] | 1.15 [1.08, 1.20] | 1.15 [1.06, 1.21] | 0.71 | nonnorm |
| Vascular Fractal Dimension in the Superior Disk Region (median [IQR]) |  | 1.40 [1.37, 1.42] | 1.40 [1.38, 1.42] | 1.40 [1.37, 1.42] | 0.454 | nonnorm |
| Vascular Fractal Dimension in the Temporal Disk Region (median [IQR]) |  | 1.38 [1.34, 1.41] | 1.38 [1.35, 1.41] | 1.37 [1.33, 1.41] | 0.074 | nonnorm |
| Vascular Length (unit: μm) (median [IQR]) |  | 1352.05 [1210.38, 1527.51] | 1377.51 [1233.28, 1550.28] | 1337.99 [1197.97, 1515.06] | 0.126 | nonnorm |
| Average Vascular Diameter (unit: μm) (median [IQR]) |  | 74.57 [69.17, 82.56] | 74.58 [70.24, 81.12] | 74.40 [68.72, 83.24] | 0.448 | nonnorm |
| Average Vascular Diameter between 0.5-1.0 PD (median [IQR]) |  | 91.56 [85.44, 98.93] | 91.96 [86.26, 98.17] | 91.20 [84.79, 99.36] | 0.252 | nonnorm |
| Average Vascular Diameter between 1.0-1.5 PD (median [IQR]) |  | 87.34 [81.24, 95.14] | 87.31 [82.32, 94.99] | 87.34 [80.58, 95.65] | 0.496 | nonnorm |
| Average Vascular Diameter between 1.5-2.0 PD (median [IQR]) |  | 82.88 [76.90, 91.30] | 83.94 [77.87, 90.86] | 82.29 [76.54, 91.45] | 0.281 | nonnorm |
| Average Vascular Diameter between 2.0-2.5 PD (median [IQR]) |  | 79.50 [73.77, 87.88] | 80.56 [73.91, 87.86] | 79.04 [73.65, 88.04] | 0.537 | nonnorm |
| Average Diameter within 3mm of Fovea Centralis (median [IQR]) |  | 56.47 [52.00, 62.24] | 56.54 [53.14, 62.26] | 56.44 [51.31, 62.24] | 0.217 | nonnorm |
| Average Diameter within 5mm of Fovea Centralis (median [IQR]) |  | 75.15 [69.63, 82.90] | 75.25 [70.46, 81.75] | 75.11 [69.06, 83.52] | 0.909 | nonnorm |
| Average Diameter in the Superior Disk Region (median [IQR]) |  | 91.98 [85.38, 99.37] | 92.37 [87.28, 99.52] | 91.59 [84.62, 99.13] | 0.108 | nonnorm |
| Average Diameter in the Temporal Disk Region (median [IQR]) |  | 65.13 [60.63, 71.35] | 65.37 [61.47, 71.28] | 65.11 [59.99, 71.35] | 0.268 | nonnorm |
| Average Diameter in the Inferior Disk Region (median [IQR]) |  | 94.73 [87.68, 103.12] | 95.48 [88.63, 104.22] | 94.40 [87.42, 102.94] | 0.347 | nonnorm |
| Average Diameter in the Nasal Disk Region (mean (SD)) |  | 75.69 (11.72) | 75.41 (11.25) | 75.83 (11.97) | 0.653 |  |
| Average Vascular Curvature (median [IQR]) |  | 0.86 [0.80, 0.95] | 0.87 [0.80, 0.97] | 0.86 [0.79, 0.95] | 0.324 | nonnorm |
| Average Vascular Curvature between 0.5-1.0 PD (median [IQR]) |  | 0.82 [0.73, 0.91] | 0.82 [0.72, 0.91] | 0.82 [0.73, 0.92] | 0.729 | nonnorm |
| Average Vascular Curvature between 1.0-1.5 PD (median [IQR]) |  | 0.78 [0.69, 0.88] | 0.80 [0.69, 0.90] | 0.77 [0.69, 0.88] | 0.149 | nonnorm |
| Average Vascular Curvature between 1.5-2.0 PD (median [IQR]) |  | 0.83 [0.73, 0.95] | 0.84 [0.73, 0.95] | 0.82 [0.72, 0.95] | 0.732 | nonnorm |
| Average Vascular Curvature between 2.0-2.5 PD (median [IQR]) |  | 0.84 [0.75, 0.97] | 0.85 [0.75, 0.99] | 0.84 [0.76, 0.96] | 0.484 | nonnorm |
| Vascular Curvature within 3mm of Fovea Centralis (median [IQR]) |  | 1.11 [0.98, 1.27] | 1.12 [0.99, 1.29] | 1.11 [0.98, 1.26] | 0.244 | nonnorm |
| Vascular Curvature within 5mm of Fovea Centralis (median [IQR]) |  | 0.92 [0.83, 1.03] | 0.93 [0.84, 1.04] | 0.91 [0.83, 1.02] | 0.247 | nonnorm |
| Vascular Curvature in the Superior Disk Region (median [IQR]) |  | 0.72 [0.65, 0.80] | 0.72 [0.66, 0.80] | 0.72 [0.65, 0.80] | 0.965 | nonnorm |
| Vascular Curvature in the Temporal Disk Region (median [IQR]) |  | 0.98 [0.89, 1.09] | 0.98 [0.89, 1.10] | 0.98 [0.89, 1.08] | 0.378 | nonnorm |
| Vascular Curvature in the Inferior Disk Region (median [IQR]) |  | 0.75 [0.68, 0.85] | 0.76 [0.68, 0.87] | 0.74 [0.68, 0.84] | 0.328 | nonnorm |
| Vascular Curvature in the Nasal Disk Region (median [IQR]) |  | 0.77 [0.65, 0.93] | 0.77 [0.64, 0.92] | 0.78 [0.65, 0.94] | 0.958 | nonnorm |
| Average Arterial Curvature (median [IQR]) |  | 0.83 [0.74, 0.96] | 0.84 [0.73, 0.98] | 0.82 [0.75, 0.96] | 0.505 | nonnorm |
| Average Arterial Curvature between 0.5-1.0 PD (median [IQR]) |  | 0.77 [0.65, 0.92] | 0.75 [0.64, 0.91] | 0.78 [0.65, 0.93] | 0.301 | nonnorm |
| Average Arterial Curvature between 1.0-1.5 PD (median [IQR]) |  | 0.78 [0.63, 0.94] | 0.81 [0.63, 0.98] | 0.76 [0.64, 0.93] | 0.255 | nonnorm |
| Average Arterial Curvature between 1.5-2.0 PD (median [IQR]) |  | 0.82 [0.68, 1.02] | 0.83 [0.68, 1.05] | 0.81 [0.69, 1.00] | 0.534 | nonnorm |
| Average Arterial Curvature between 2.0-2.5 PD (median [IQR]) |  | 0.83 [0.70, 1.00] | 0.84 [0.70, 1.00] | 0.83 [0.70, 0.99] | 0.693 | nonnorm |
| Arterial Curvature within 3mm of Fovea Centralis (median [IQR]) |  | 1.02 [0.85, 1.25] | 1.03 [0.85, 1.27] | 1.02 [0.85, 1.22] | 0.594 | nonnorm |
| Arterial Curvature within 5mm of Fovea Centralis (median [IQR]) |  | 0.88 [0.77, 1.02] | 0.88 [0.77, 1.05] | 0.89 [0.78, 1.01] | 0.671 | nonnorm |
| Arterial Curvature in the Superior Disk Region (median [IQR]) |  | 0.71 [0.61, 0.82] | 0.71 [0.60, 0.82] | 0.71 [0.62, 0.82] | 0.877 | nonnorm |
| Arterial Curvature in the Temporal Disk Region (median [IQR]) |  | 0.93 [0.82, 1.10] | 0.92 [0.82, 1.10] | 0.93 [0.82, 1.10] | 0.967 | nonnorm |
| Arterial Curvature in the Inferior Disk Region (median [IQR]) |  | 0.76 [0.64, 0.90] | 0.78 [0.64, 0.96] | 0.76 [0.64, 0.88] | 0.199 | nonnorm |
| Arterial Curvature in the Nasal Disk Region (median [IQR]) |  | 0.74 [0.58, 0.93] | 0.74 [0.58, 0.93] | 0.74 [0.58, 0.94] | 0.971 | nonnorm |
| Average Venous Curvature (median [IQR]) |  | 0.89 [0.83, 0.96] | 0.89 [0.83, 0.96] | 0.89 [0.82, 0.96] | 0.467 | nonnorm |
| Average Venous Curvature between 0.5-1.0 PD (median [IQR]) |  | 0.84 [0.74, 0.95] | 0.85 [0.76, 0.95] | 0.83 [0.73, 0.95] | 0.463 | nonnorm |
| Average Venous Curvature between 1.0-1.5 PD (median [IQR]) |  | 0.76 [0.68, 0.87] | 0.78 [0.68, 0.88] | 0.75 [0.68, 0.87] | 0.33 | nonnorm |
| Average Venous Curvature between 1.5-2.0 PD (median [IQR]) |  | 0.80 [0.70, 0.91] | 0.80 [0.71, 0.91] | 0.80 [0.70, 0.92] | 0.885 | nonnorm |
| Average Venous Curvature between 2.0-2.5 PD (median [IQR]) |  | 0.84 [0.73, 0.98] | 0.83 [0.73, 0.99] | 0.84 [0.74, 0.97] | 0.734 | nonnorm |
| Venous Curvature within 3mm of Fovea Centralis (median [IQR]) |  | 1.19 [1.06, 1.35] | 1.22 [1.06, 1.37] | 1.19 [1.06, 1.34] | 0.125 | nonnorm |
| Venous Curvature within 5mm of Fovea Centralis (median [IQR]) |  | 0.95 [0.86, 1.05] | 0.95 [0.87, 1.06] | 0.95 [0.86, 1.04] | 0.173 | nonnorm |
| Venous Curvature in the Superior Disk Region (median [IQR]) |  | 0.72 [0.65, 0.79] | 0.72 [0.66, 0.79] | 0.73 [0.65, 0.79] | 0.907 | nonnorm |
| Venous Curvature in the Temporal Disk Region (median [IQR]) |  | 1.01 [0.92, 1.12] | 1.03 [0.93, 1.12] | 1.00 [0.92, 1.12] | 0.11 | nonnorm |
| Venous Curvature in the Inferior Disk Region (median [IQR]) |  | 0.73 [0.65, 0.82] | 0.72 [0.66, 0.80] | 0.73 [0.65, 0.82] | 0.638 | nonnorm |
| Venous Curvature in the Nasal Disk Region (median [IQR]) |  | 0.78 [0.64, 0.95] | 0.77 [0.64, 0.93] | 0.79 [0.65, 0.97] | 0.254 | nonnorm |
| Vascular Density (median [IQR]) |  | 0.08 [0.07, 0.09] | 0.09 [0.08, 0.10] | 0.08 [0.07, 0.09] | <0.001 | nonnorm |
| Vascular Coverage Density between 0.5-1.0 PD (median [IQR]) |  | 0.14 [0.13, 0.15] | 0.14 [0.13, 0.15] | 0.14 [0.12, 0.15] | <0.001 | nonnorm |
| Vascular Coverage Density between 1.0-1.5 PD (median [IQR]) |  | 0.10 [0.09, 0.11] | 0.10 [0.09, 0.11] | 0.10 [0.09, 0.11] | 0.055 | nonnorm |
| Vascular Coverage Density between 1.5-2.0 PD (median [IQR]) |  | 0.07 [0.06, 0.08] | 0.07 [0.06, 0.08] | 0.07 [0.06, 0.08] | 0.196 | nonnorm |
| Vascular Coverage Density between 2.0-2.5 PD (median [IQR]) |  | 0.05 [0.04, 0.06] | 0.06 [0.05, 0.06] | 0.05 [0.04, 0.06] | 0.016 | nonnorm |
| Average Vascular Density within 3mm of Fovea Centralis (median [IQR]) |  | 0.05 [0.04, 0.06] | 0.05 [0.04, 0.06] | 0.05 [0.04, 0.06] | 0.047 | nonnorm |
| Average Vascular Density within 5mm of Fovea Centralis (median [IQR]) |  | 0.07 [0.06, 0.08] | 0.07 [0.07, 0.08] | 0.07 [0.06, 0.08] | 0.091 | nonnorm |
| Average Vascular Density in the Superior Region (median [IQR]) |  | 0.12 [0.11, 0.14] | 0.12 [0.11, 0.14] | 0.12 [0.11, 0.14] | 0.237 | nonnorm |
| Average Vascular Density in the Temporal Region (median [IQR]) |  | 0.06 [0.05, 0.07] | 0.06 [0.05, 0.07] | 0.06 [0.05, 0.07] | 0.127 | nonnorm |
| Average Vascular Density in the Inferior Region (median [IQR]) |  | 0.11 [0.09, 0.13] | 0.12 [0.11, 0.14] | 0.10 [0.08, 0.12] | <0.001 | nonnorm |
| Average Vascular Density in the Nasal Region (median [IQR]) |  | 0.07 [0.05, 0.09] | 0.07 [0.05, 0.09] | 0.07 [0.05, 0.09] | 0.705 | nonnorm |
| Average Arterial Diameter (unit: μm) (median [IQR]) |  | 68.50 [63.29, 75.25] | 69.00 [64.10, 74.62] | 68.26 [63.01, 75.61] | 0.545 | nonnorm |
| Average Arterial Diameter between 0.5-1.0 PD (median [IQR]) |  | 78.05 [72.04, 85.24] | 78.59 [72.81, 84.93] | 77.75 [71.54, 85.45] | 0.476 | nonnorm |
| Average Arterial Diameter between 1.5-2.0 PD (median [IQR]) |  | 74.71 [67.88, 81.46] | 74.94 [69.23, 82.17] | 74.60 [67.39, 81.01] | 0.152 | nonnorm |
| Average Arterial Diameter between 2.0-2.5 PD (median [IQR]) |  | 71.07 [65.35, 77.99] | 71.76 [65.56, 77.31] | 70.64 [65.24, 78.28] | 0.435 | nonnorm |
| Arterial Diameter within 3mm of Fovea Centralis (median [IQR]) |  | 69.74 [63.26, 76.10] | 69.66 [64.05, 75.64] | 69.76 [63.04, 76.51] | 0.936 | nonnorm |
| Arterial Diameter within 5mm of Fovea Centralis (median [IQR]) |  | 55.21 [49.27, 61.46] | 55.61 [49.95, 60.62] | 54.95 [49.20, 61.72] | 0.583 | nonnorm |
| Arterial Diameter in the Superior Disk Region (median [IQR]) |  | 67.28 [62.26, 74.05] | 67.43 [62.50, 72.79] | 67.24 [61.93, 74.36] | 0.636 | nonnorm |
| Arterial Diameter in the Temporal Disk Region (median [IQR]) |  | 77.31 [71.24, 84.19] | 77.40 [71.65, 84.48] | 77.25 [71.01, 83.97] | 0.55 | nonnorm |
| Arterial Diameter in the Inferior Disk Region (median [IQR]) |  | 59.16 [54.67, 64.91] | 60.25 [55.73, 64.88] | 58.79 [54.18, 64.96] | 0.128 | nonnorm |
| Arterial Diameter in the Nasal Disk Region (median [IQR]) |  | 78.42 [71.91, 85.81] | 78.57 [72.96, 85.76] | 78.39 [71.12, 85.81] | 0.434 | nonnorm |
| Average Venous Diameter (unit: μm) (mean (SD)) |  | 59.70 (12.02) | 59.22 (11.47) | 59.95 (12.30) | 0.454 |  |
| Average Venous Diameter between 0.5-1.0 PD (median [IQR]) |  | 68.50 [63.29, 75.25] | 69.00 [64.10, 74.62] | 68.26 [63.01, 75.61] | 0.545 | nonnorm |
| Average Venous Diameter between 1.0-1.5 PD (median [IQR]) |  | 103.60 [95.16, 112.51] | 103.46 [97.96, 110.99] | 103.81 [94.56, 112.97] | 0.737 | nonnorm |
| Average Venous Diameter between 1.5-2.0 PD (median [IQR]) |  | 101.21 [93.26, 110.66] | 99.81 [93.82, 109.83] | 101.63 [93.19, 111.10] | 0.347 | nonnorm |
| Average Venous Diameter between 2.0-2.5 PD (median [IQR]) |  | 96.13 [88.06, 106.89] | 97.33 [89.42, 107.17] | 94.90 [87.54, 106.74] | 0.227 | nonnorm |
| Venous Diameter within 3mm of Fovea Centralis (median [IQR]) |  | 91.68 [83.46, 103.79] | 92.60 [83.12, 104.12] | 91.13 [83.70, 103.13] | 0.568 | nonnorm |
| Venous Diameter within 5mm of Fovea Centralis (median [IQR]) |  | 57.45 [52.95, 63.95] | 58.38 [53.43, 65.05] | 56.97 [52.34, 63.22] | 0.054 | nonnorm |
| Venous Diameter in the Superior Disk Region (median [IQR]) |  | 84.07 [76.65, 92.74] | 83.64 [77.37, 91.73] | 84.47 [76.17, 93.07] | 0.712 | nonnorm |
| Venous Diameter in the Temporal Disk Region (median [IQR]) |  | 107.74 [99.38, 116.67] | 109.27 [100.57, 116.88] | 107.41 [98.27, 116.46] | 0.141 | nonnorm |
| Venous Diameter in the Inferior Disk Region (median [IQR]) |  | 71.31 [65.13, 78.68] | 71.12 [66.29, 78.70] | 71.34 [64.78, 78.65] | 0.494 | nonnorm |
| Venous Diameter in the Nasal Disk Region (median [IQR]) |  | 113.66 [103.72, 124.66] | 114.92 [104.73, 125.27] | 112.96 [103.67, 122.84] | 0.397 | nonnorm |
| Arterial to Venous Diameter Ratio (mean (SD)) |  | 89.44 (16.79) | 88.75 (16.17) | 89.79 (17.11) | 0.438 |  |
| Angle between Vascular Arc and Fovea (unit: degrees) (median [IQR]) |  | 0.79 [0.74, 0.85] | 0.80 [0.75, 0.86] | 0.79 [0.74, 0.85] | 0.54 | nonnorm |
| Distance between Vascular Arc and Fovea Intersection (unit: μm) (median [IQR]) |  | 85.94 [80.57, 90.63] | 85.85 [80.61, 91.09] | 85.97 [80.57, 90.47] | 0.865 | nonnorm |
| Angle between Vascular Arc and 1PD Outside Disk (unit: degrees) (median [IQR]) |  | 9259.48 [8433.10, 9989.62] | 9312.58 [8456.33, 10054.34] | 9229.61 [8399.91, 9966.39] | 0.19 | nonnorm |
| Distance between Vascular Arc and 1PD Outside Disk Intersection (unit: μm) (median [IQR]) |  | 129.94 [124.10, 134.69] | 130.44 [124.59, 135.20] | 129.64 [123.90, 134.47] | 0.388 | nonnorm |
| Angle between Vascular Arc and 2PD Outside Disk (unit: degrees) (median [IQR]) |  | 3710.43 [3538.23, 3867.87] | 3718.85 [3543.76, 3899.86] | 3706.19 [3538.12, 3859.78] | 0.793 | nonnorm |
| Distance between Vascular Arc and 2PD Outside Disk Intersection (unit: μm) (median [IQR]) |  | 111.77 [104.70, 117.35] | 111.91 [105.01, 118.27] | 111.59 [104.58, 116.95] | 0.377 | nonnorm |
| Optic Disk Area (unit: mm) (mean (SD)) |  | 6757.26 (531.45) | 6759.45 (545.67) | 6756.13 (524.55) | 0.938 |  |
| Horizontal Diameter of Optic Disk (unit: μm) (median [IQR]) |  | 2.79 [2.48, 3.15] | 2.80 [2.47, 3.17] | 2.78 [2.50, 3.11] | 0.894 | nonnorm |
| Vertical Diameter of Optic Disk (unit: μm) (median [IQR]) |  | 1789.99 [1680.15, 1912.03] | 1785.92 [1674.97, 1913.05] | 1794.05 [1681.81, 1912.03] | 0.524 | nonnorm |
| Optic Disk Roundness (median [IQR]) |  | 1981.19 [1863.21, 2111.49] | 1985.26 [1857.11, 2125.12] | 1981.19 [1867.28, 2107.30] | 0.88 | nonnorm |
| Optic Disk Tilt Angle (unit: degrees) (median [IQR]) |  | 0.86 [0.82, 0.89] | 0.85 [0.82, 0.89] | 0.86 [0.82, 0.89] | 0.517 | nonnorm |
| Distance from Center of Optic Disk to Boundary (unit: μm) (median [IQR]) |  | -49.71 [-77.64, 75.40] | -54.34 [-77.34, 76.26] | -48.19 [-77.64, 75.34] | 0.826 | nonnorm |
| Distance from Optic Disk to Fovea (unit: μm) (median [IQR]) |  | 3194.20 [2962.90, 3408.49] | 3188.49 [2994.76, 3402.21] | 3194.35 [2957.76, 3412.26] | 0.637 | nonnorm |
| Angle between Line Connecting Fovea and Optic Disk (unit: degrees) (median [IQR]) |  | 4978.44 [4781.11, 5145.04] | 5011.14 [4810.17, 5165.91] | 4964.53 [4760.75, 5136.06] | 0.048 | nonnorm |
| Ratio of Long Axis to Short Axis of Optic Disk (mean (SD)) |  | 6.14 (3.59) | 6.36 (3.67) | 6.02 (3.54) | 0.233 |  |
| Distance from Center of Fovea to Boundary (unit: μm) (median [IQR]) |  | 1.13 [1.09, 1.18] | 1.14 [1.09, 1.18] | 1.13 [1.09, 1.18] | 0.097 | nonnorm |
| Cup Area (unit: mm) (median [IQR]) |  | 3897.17 [3803.67, 4066.45] | 3924.96 [3812.38, 4041.76] | 3889.02 [3801.07, 4084.36] | 0.381 | nonnorm |
| Horizontal Diameter of Optic Cup (unit: μm) (median [IQR]) |  | 0.67 [0.51, 0.86] | 0.68 [0.50, 0.87] | 0.67 [0.51, 0.86] | 0.806 | nonnorm |
| Vertical Diameter of Optic Cup (unit: μm) (median [IQR]) |  | 911.27 [781.09, 1053.65] | 915.34 [784.14, 1053.65] | 911.27 [781.09, 1050.62] | 0.842 | nonnorm |
| Optic Cup Roundness (median [IQR]) |  | 925.31 [813.63, 1037.38] | 933.16 [805.49, 1037.38] | 923.47 [817.70, 1033.31] | 0.852 | nonnorm |
| Area Cup to Disk Ratio (median [IQR]) |  | 0.89 [0.86, 0.91] | 0.89 [0.86, 0.91] | 0.89 [0.85, 0.91] | 0.461 | nonnorm |
| Horizontal Cup to Disk Ratio (median [IQR]) |  | 0.24 [0.20, 0.30] | 0.25 [0.20, 0.30] | 0.24 [0.20, 0.29] | 0.598 | nonnorm |
| Vertical Cup to Disk Ratio (median [IQR]) |  | 0.52 [0.45, 0.57] | 0.52 [0.45, 0.57] | 0.51 [0.45, 0.57] | 0.431 | nonnorm |
| Disk Rim I Distance (unit: μm) (median [IQR]) |  | 0.47 [0.43, 0.51] | 0.47 [0.42, 0.51] | 0.46 [0.43, 0.51] | 0.757 | nonnorm |
| Disk Rim N Distance (unit: μm) (median [IQR]) |  | 524.79 [475.97, 573.61] | 520.72 [471.91, 566.49] | 528.86 [480.04, 573.61] | 0.331 | nonnorm |
| Disk Rim S Distance (unit: μm). (median [IQR]) |  | 402.75 [353.93, 455.63] | 406.81 [358.00, 455.63] | 402.75 [353.70, 455.63] | 0.62 | nonnorm |
| Disk Rim T Distance (unit: μm) (median [IQR]) |  | 528.86 [475.97, 581.75] | 528.86 [475.97, 581.75] | 528.86 [475.97, 581.75] | 0.41 | nonnorm |
| Vertical to Horizontal Ratio of Optic Disk Vascular Arc Direction (median [IQR]) |  | 455.63 [378.09, 532.93] | 435.72 [374.27, 516.65] | 459.70 [378.34, 541.06] | 0.09 | nonnorm |
| Optic Disk Short to Long Axis Ratio (median [IQR]) |  | 1.11 [1.05, 1.16] | 1.12 [1.06, 1.17] | 1.10 [1.05, 1.16] | 0.127 | nonnorm |
| Angle between Optic Disk Long Axis and Vertical Line (median [IQR]) |  | 0.88 [0.85, 0.92] | 0.88 [0.85, 0.91] | 0.89 [0.85, 0.92] | 0.091 | nonnorm |
| Maximum Rim Distance between Optic Disk and Optic Cup (median [IQR]) |  | -1.95 [-14.94, 11.27] | -2.12 [-15.41, 9.01] | -1.95 [-14.74, 12.12] | 0.348 | nonnorm |
| Minimum Rim Distance between Optic Disk and Optic Cup (median [IQR]) |  | 1540.41 [1429.68, 1652.18] | 1547.41 [1419.88, 1652.26] | 1537.34 [1432.32, 1652.18] | 0.922 | nonnorm |
| Minimum Rim Distance between Optic Disk and Optic Cup 1 (median [IQR]) |  | 350.07 [308.24, 397.64] | 352.64 [308.13, 398.26] | 348.87 [308.30, 394.11] | 0.771 | nonnorm |
| Angle between Minimum Rim of Optic Disk and Horizontal Line Start (median [IQR]) |  | -18.64 [-145.31, 11.07] | -18.70 [-145.37, 10.88] | -18.64 [-145.13, 11.07] | 0.761 | nonnorm |
| Angle between Minimum Rim of Optic Disk and Horizontal Line End (median [IQR]) |  | -18.26 [-145.31, 11.77] | -17.86 [-145.37, 12.91] | -18.64 [-145.13, 11.07] | 0.948 | nonnorm |
| Angle between Line Connecting Fovea and Optic Disk and Long Axis of Optic Disk (median [IQR]) |  | 71.26 [60.13, 78.65] | 71.57 [60.91, 78.69] | 71.20 [59.88, 78.64] | 0.395 | nonnorm |
| Arterial Centerline Fractal Dimension (median [IQR]) |  | 1.26 [1.23, 1.29] | 1.26 [1.24, 1.29] | 1.26 [1.22, 1.29] | 0.31 | nonnorm |
| Vascular Centerline Fractal Dimension (median [IQR]) |  | 1.41 [1.38, 1.43] | 1.41 [1.39, 1.43] | 1.41 [1.37, 1.43] | 0.22 | nonnorm |
| Venous Centerline Fractal Dimension (median [IQR]) |  | 1.27 [1.24, 1.29] | 1.27 [1.25, 1.29] | 1.27 [1.24, 1.29] | 0.063 | nonnorm |
| Retinal Arterial Density (median [IQR]) |  | 0.04 [0.03, 0.04] | 0.04 [0.03, 0.04] | 0.04 [0.03, 0.04] | 0.019 | nonnorm |
| Retinal Venous Density (median [IQR]) |  | 0.05 [0.04, 0.05] | 0.06 [0.05, 0.07] | 0.05 [0.03, 0.05] | <0.001 | nonnorm |
| Arterial Fractal Dimension (median [IQR]) |  | 1.39 [1.36, 1.41] | 1.40 [1.37, 1.42] | 1.39 [1.35, 1.41] | 0.106 | nonnorm |
| Venous Fractal Dimension (median [IQR]) |  | 1.43 [1.40, 1.45] | 1.45 [1.43, 1.47] | 1.42 [1.38, 1.44] | <0.001 | nonnorm |
| Arterial Vessel Length (median [IQR]) |  | 714.75 [626.01, 827.44] | 733.15 [632.30, 841.20] | 704.16 [624.03, 821.03] | 0.165 | nonnorm |
| Venous Vessel Length (median [IQR]) |  | 627.93 [559.84, 718.83] | 643.05 [562.26, 727.98] | 625.87 [559.07, 713.44] | 0.258 | nonnorm |
| Main Arterial.Venous Ratio in Vascular Arc (median [IQR]) |  | 0.70 [0.64, 0.78] | 0.70 [0.63, 0.77] | 0.70 [0.64, 0.78] | 0.984 | nonnorm |
| Main Arterial.Venous Ratio in Lower Vascular Arc (median [IQR]) |  | 0.63 [0.57, 0.71] | 0.63 [0.56, 0.71] | 0.64 [0.57, 0.71] | 0.573 | nonnorm |
| Main Arterial.Venous Ratio in Upper Vascular Arc (median [IQR]) |  | 0.66 [0.60, 0.73] | 0.67 [0.61, 0.74] | 0.66 [0.60, 0.73] | 0.249 | nonnorm |
| Average Arterial Branching Angle (median [IQR]) |  | 64.97 [57.71, 70.77] | 65.17 [58.74, 72.56] | 64.77 [57.20, 69.78] | 0.039 | nonnorm |
| Average Venous Branching Angle (median [IQR]) |  | 64.59 [58.75, 69.69] | 64.72 [59.25, 70.07] | 64.44 [58.50, 69.38] | 0.55 | nonnorm |
| Distance from Nasal Edge of Optic Disk to Fovea Center (unit: μm) (median [IQR]) |  | 5864.29 [5653.09, 6048.46] | 5898.07 [5699.12, 6063.15] | 5850.24 [5634.94, 6034.12] | 0.065 | nonnorm |
| Arterial Vessel Area (median [IQR]) |  | 4.25 [3.66, 4.76] | 4.29 [3.79, 4.81] | 4.20 [3.59, 4.72] | 0.049 | nonnorm |
| Venous Vessel Area (median [IQR]) |  | 5.94 [5.37, 6.40] | 6.23 [5.66, 6.67] | 5.77 [5.16, 6.25] | <0.001 | nonnorm |
| Vascular Area (median [IQR]) |  | 10.19 [9.29, 10.89] | 10.59 [9.81, 11.33] | 9.93 [9.10, 10.76] | <0.001 | nonnorm |
| Vascular Area within 0.5-1.0 PD (mean (SD)) |  | 1.38 (0.24) | 1.40 (0.24) | 1.37 (0.24) | 0.103 |  |
| Vascular Area within 1.0-1.5 PD (mean (SD)) |  | 1.63 (0.31) | 1.67 (0.32) | 1.61 (0.30) | 0.039 |  |
| Vascular Area within 1.5-2.0 PD (median [IQR]) |  | 1.54 [1.31, 1.75] | 1.58 [1.33, 1.74] | 1.53 [1.30, 1.75] | 0.406 | nonnorm |
| Vascular Area within 2.0-2.5 PD (median [IQR]) |  | 1.37 [1.24, 1.54] | 1.51 [1.33, 1.66] | 1.32 [1.15, 1.47] | <0.001 | nonnorm |

**S3 Results of single-factor and multiple-factor regression**

| name | desc | 0 (N=189) | 1 (N=366) | OR (univariable) | OR (multivariable) |
| --- | --- | --- | --- | --- | --- |
| sex | 0 | 113 (59.8%) | 166 (45.4%) |  |  |
| Presence of Leopard Spot Lesions | 0 | 144 (76.2%) | 140 (38.3%) |  |  |
| Vascular Fractal Dimension | Mean ± SD | 1.6 ± 0.0 | 1.5 ± 0.0 | 0.00 (0.00-0.00, p<.001) | 0.00 (0.00-0.00, p<.001) |
| Vascular Coverage Density between 0.5-1.0 PD | Mean ± SD | 0.1 ± 0.0 | 0.1 ± 0.0 | 0.00 (0.00-0.00, p<.001) | 0.00 (0.00-0.00, p<.001) |
| Vascular Coverage Density between 2.0-2.5 PD | Mean ± SD | 0.1 ± 0.0 | 0.1 ± 0.0 | 0.00 (0.00-0.63, p=.043) | 4758372422945087579110082653192192.00 (3205269722583626.00-7064025830934859015488922918641682270052866511077376.00, p<.001) |
| Average Vascular Density in the Inferior Region | Mean ± SD | 0.1 ± 0.0 | 0.1 ± 0.0 | 0.00 (0.00-0.00, p<.001) | 0.00 (0.00-0.00, p=.002) |
| Venous Fractal Dimension | Mean ± SD | 1.5 ± 0.0 | 1.4 ± 0.1 | 0.00 (0.00-0.00, p<.001) | 0.00 (0.00-0.00, p<.001) |
| Venous Vessel Area | Mean ± SD | 6.2 ± 0.8 | 5.7 ± 0.8 | 0.36 (0.27-0.47, p<.001) | 14.83 (2.19-100.37, p=.006) |
| Vascular Area within 1.0-1.5 PD | Mean ± SD | 1.7 ± 0.3 | 1.6 ± 0.3 | 0.55 (0.31-0.98, p=.044) | 34.87 (3.79-320.42, p=.002) |
| Vascular Area within 2.0-2.5 PD | Mean ± SD | 1.5 ± 0.2 | 1.3 ± 0.2 | 0.01 (0.00-0.03, p<.001) | 0.00 (0.00-0.01, p<.001) |
|  | 1 | 76 (40.2%) | 200 (54.6%) | 1.79 (1.25-2.56, p=.001) | 3.06 (1.29-7.24, p=.011) |
| Presence of Atrophic Regions | 0 | 134 (70.9%) | 257 (70.2%) |  |  |
|  | 1 | 55 (29.1%) | 109 (29.8%) | 1.03 (0.70-1.52, p=.868) |  |
|  | 1 | 45 (23.8%) | 226 (61.7%) | 5.17 (3.48-7.67, p<.001) | 10.95 (4.81-24.92, p<.001) |
| Age | Mean ± SD | 7.8 ± 1.0 | 7.7 ± 0.9 | 0.95 (0.79-1.15, p=.589) |  |
| DS | Mean ± SD | 0.8 ± 1.1 | 0.4 ± 1.1 | 0.67 (0.56-0.81, p<.001) | 0.37 (0.11-1.25, p=.110) |
| AL | Mean ± SD | 23.1 ± 0.8 | 23.2 ± 0.8 | 1.25 (1.00-1.56, p=.049) | 0.82 (0.44-1.50, p=.516) |
| SE | Mean ± SD | 0.4 ± 1.2 | 0.1 ± 1.1 | 0.74 (0.62-0.88, p<.001) | 2.31 (0.74-7.24, p=.149) |
| Average Vascular Branching Angle | Mean ± SD | 64.0 ± 6.3 | 63.4 ± 7.0 | 0.99 (0.96-1.01, p=.262) |  |
| Vascular Fractal Dimension in the Inferior Disk Region | Mean ± SD | 1.4 ± 0.0 | 1.3 ± 0.0 | 0.00 (0.00-0.00, p<.001) | 23.18 (0.00-140609113.06, p=.693) |
| Vascular Fractal Dimension in the Nasal Disk Region | Mean ± SD | 1.1 ± 0.1 | 1.1 ± 0.1 | 0.39 (0.07-2.05, p=.267) |  |
| Vascular Fractal Dimension in the Superior Disk Region | Mean ± SD | 1.4 ± 0.0 | 1.4 ± 0.0 | 0.24 (0.00-18.10, p=.516) |  |
| Vascular Fractal Dimension in the Temporal Disk Region | Mean ± SD | 1.4 ± 0.1 | 1.4 ± 0.1 | 0.07 (0.00-1.18, p=.065) |  |
| Vascular Length (unit: μm) | Mean ± SD | 1417.4 ± 249.8 | 1399.2 ± 280.2 | 1.00 (1.00-1.00, p=.451) |  |
| Average Vascular Diameter (unit: μm) | Mean ± SD | 77.2 ± 9.7 | 76.8 ± 10.3 | 1.00 (0.98-1.01, p=.671) |  |
| Average Vascular Diameter between 0.5-1.0 PD | Mean ± SD | 93.4 ± 9.1 | 92.3 ± 10.0 | 0.99 (0.97-1.01, p=.199) |  |
| Average Vascular Diameter between 1.0-1.5 PD | Mean ± SD | 89.5 ± 9.4 | 88.8 ± 10.3 | 0.99 (0.98-1.01, p=.410) |  |
| Average Vascular Diameter between 1.5-2.0 PD | Mean ± SD | 85.9 ± 10.8 | 85.1 ± 11.5 | 0.99 (0.98-1.01, p=.441) |  |
| Average Vascular Diameter between 2.0-2.5 PD | Mean ± SD | 82.5 ± 11.2 | 82.2 ± 11.9 | 1.00 (0.98-1.01, p=.792) |  |
| Average Diameter within 3mm of Fovea Centralis | Mean ± SD | 58.5 ± 8.3 | 58.0 ± 8.7 | 0.99 (0.97-1.01, p=.521) |  |
| Average Diameter within 5mm of Fovea Centralis | Mean ± SD | 77.1 ± 10.0 | 77.3 ± 10.8 | 1.00 (0.99-1.02, p=.819) |  |
| Average Diameter in the Superior Disk Region | Mean ± SD | 94.5 ± 10.7 | 92.8 ± 10.7 | 0.99 (0.97-1.00, p=.072) |  |
| Average Diameter in the Temporal Disk Region | Mean ± SD | 67.5 ± 8.6 | 67.1 ± 9.4 | 0.99 (0.98-1.01, p=.593) |  |
| Average Diameter in the Inferior Disk Region | Mean ± SD | 96.9 ± 11.6 | 96.1 ± 11.6 | 0.99 (0.98-1.01, p=.490) |  |
| Average Diameter in the Nasal Disk Region | Mean ± SD | 76.3 ± 11.1 | 75.3 ± 12.0 | 0.99 (0.98-1.01, p=.353) |  |
| Average Vascular Curvature | Mean ± SD | 0.9 ± 0.1 | 0.9 ± 0.1 | 0.40 (0.09-1.73, p=.221) |  |
| Average Vascular Curvature between 0.5-1.0 PD | Mean ± SD | 0.8 ± 0.2 | 0.8 ± 0.1 | 0.94 (0.28-3.16, p=.922) |  |
| Average Vascular Curvature between 1.0-1.5 PD | Mean ± SD | 0.8 ± 0.2 | 0.8 ± 0.2 | 0.43 (0.15-1.27, p=.126) |  |
| Average Vascular Curvature between 1.5-2.0 PD | Mean ± SD | 0.9 ± 0.2 | 0.8 ± 0.2 | 0.60 (0.22-1.68, p=.333) |  |
| Average Vascular Curvature between 2.0-2.5 PD | Mean ± SD | 0.9 ± 0.2 | 0.9 ± 0.2 | 0.48 (0.17-1.38, p=.173) |  |
| Vascular Curvature within 3mm of Fovea Centralis | Mean ± SD | 1.1 ± 0.2 | 1.1 ± 0.2 | 0.60 (0.27-1.31, p=.198) |  |
| Vascular Curvature within 5mm of Fovea Centralis | Mean ± SD | 0.9 ± 0.2 | 0.9 ± 0.1 | 0.40 (0.12-1.31, p=.131) |  |
| Vascular Curvature in the Superior Disk Region | Mean ± SD | 0.7 ± 0.1 | 0.7 ± 0.1 | 0.75 (0.17-3.41, p=.715) |  |
| Vascular Curvature in the Temporal Disk Region | Mean ± SD | 1.0 ± 0.2 | 1.0 ± 0.2 | 0.51 (0.17-1.51, p=.223) |  |
| Vascular Curvature in the Inferior Disk Region | Mean ± SD | 0.8 ± 0.1 | 0.8 ± 0.1 | 0.52 (0.15-1.85, p=.314) |  |
| Vascular Curvature in the Nasal Disk Region | Mean ± SD | 0.8 ± 0.2 | 0.8 ± 0.2 | 1.18 (0.51-2.75, p=.695) |  |
| Average Arterial Curvature | Mean ± SD | 0.9 ± 0.2 | 0.9 ± 0.2 | 0.52 (0.19-1.45, p=.213) |  |
| Average Arterial Curvature between 0.5-1.0 PD | Mean ± SD | 0.8 ± 0.2 | 0.8 ± 0.2 | 1.29 (0.57-2.89, p=.541) |  |
| Average Arterial Curvature between 1.0-1.5 PD | Mean ± SD | 0.8 ± 0.3 | 0.8 ± 0.2 | 0.58 (0.28-1.21, p=.148) |  |
| Average Arterial Curvature between 1.5-2.0 PD | Mean ± SD | 0.9 ± 0.3 | 0.9 ± 0.2 | 0.64 (0.32-1.29, p=.213) |  |
| Average Arterial Curvature between 2.0-2.5 PD | Mean ± SD | 0.9 ± 0.3 | 0.9 ± 0.2 | 0.71 (0.34-1.50, p=.373) |  |
| Arterial Curvature within 3mm of Fovea Centralis | Mean ± SD | 1.1 ± 0.3 | 1.0 ± 0.3 | 0.69 (0.37-1.26, p=.227) |  |
| Arterial Curvature within 5mm of Fovea Centralis | Mean ± SD | 0.9 ± 0.2 | 0.9 ± 0.2 | 0.62 (0.25-1.49, p=.282) |  |
| Arterial Curvature in the Superior Disk Region | Mean ± SD | 0.7 ± 0.2 | 0.7 ± 0.2 | 0.78 (0.28-2.14, p=.624) |  |
| Arterial Curvature in the Temporal Disk Region | Mean ± SD | 1.0 ± 0.2 | 1.0 ± 0.2 | 0.79 (0.34-1.81, p=.572) |  |
| Arterial Curvature in the Inferior Disk Region | Mean ± SD | 0.8 ± 0.2 | 0.8 ± 0.2 | 0.61 (0.29-1.32, p=.211) |  |
| Arterial Curvature in the Nasal Disk Region | Mean ± SD | 0.8 ± 0.3 | 0.8 ± 0.3 | 0.95 (0.56-1.62, p=.859) |  |
| Average Venous Curvature | Mean ± SD | 0.9 ± 0.1 | 0.9 ± 0.1 | 0.56 (0.12-2.63, p=.458) |  |
| Average Venous Curvature between 0.5-1.0 PD | Mean ± SD | 0.9 ± 0.2 | 0.9 ± 0.2 | 0.62 (0.21-1.86, p=.391) |  |
| Average Venous Curvature between 1.0-1.5 PD | Mean ± SD | 0.8 ± 0.2 | 0.8 ± 0.2 | 0.60 (0.21-1.70, p=.336) |  |
| Average Venous Curvature between 1.5-2.0 PD | Mean ± SD | 0.8 ± 0.2 | 0.8 ± 0.2 | 1.02 (0.38-2.77, p=.967) |  |
| Average Venous Curvature between 2.0-2.5 PD | Mean ± SD | 0.9 ± 0.2 | 0.9 ± 0.2 | 0.59 (0.23-1.51, p=.269) |  |
| Venous Curvature within 3mm of Fovea Centralis | Mean ± SD | 1.2 ± 0.2 | 1.2 ± 0.2 | 0.61 (0.29-1.28, p=.193) |  |
| Venous Curvature within 5mm of Fovea Centralis | Mean ± SD | 1.0 ± 0.1 | 1.0 ± 0.1 | 0.37 (0.11-1.21, p=.099) |  |
| Venous Curvature in the Superior Disk Region | Mean ± SD | 0.7 ± 0.1 | 0.7 ± 0.1 | 1.03 (0.26-4.10, p=.971) |  |
| Venous Curvature in the Temporal Disk Region | Mean ± SD | 1.0 ± 0.2 | 1.0 ± 0.2 | 0.43 (0.15-1.20, p=.108) |  |
| Venous Curvature in the Inferior Disk Region | Mean ± SD | 0.7 ± 0.1 | 0.7 ± 0.1 | 0.95 (0.23-3.84, p=.942) |  |
| Venous Curvature in the Nasal Disk Region | Mean ± SD | 0.8 ± 0.3 | 0.9 ± 0.3 | 1.61 (0.86-3.02, p=.139) |  |
| Vascular Density | Mean ± SD | 0.1 ± 0.0 | 0.1 ± 0.0 | 0.00 (0.00-0.00, p<.001) | 0.00 (0.00-2595892755.06, p=.352) |
| Vascular Coverage Density between 1.0-1.5 PD | Mean ± SD | 0.1 ± 0.0 | 0.1 ± 0.0 | 0.00 (0.00-7.50, p=.108) |  |
| Vascular Coverage Density between 1.5-2.0 PD | Mean ± SD | 0.1 ± 0.0 | 0.1 ± 0.0 | 0.01 (0.00-699.22, p=.393) |  |
| Average Vascular Density within 3mm of Fovea Centralis | Mean ± SD | 0.0 ± 0.0 | 0.0 ± 0.0 | 0.00 (0.00-33.71, p=.167) |  |
| Average Vascular Density within 5mm of Fovea Centralis | Mean ± SD | 0.1 ± 0.0 | 0.1 ± 0.0 | 0.00 (0.00-12.17, p=.118) |  |
| Average Vascular Density in the Superior Region | Mean ± SD | 0.1 ± 0.0 | 0.1 ± 0.0 | 0.01 (0.00-52.89, p=.276) |  |
| Average Vascular Density in the Temporal Region | Mean ± SD | 0.1 ± 0.0 | 0.1 ± 0.0 | 0.00 (0.00-33.23, p=.168) |  |
| Average Vascular Density in the Nasal Region | Mean ± SD | 0.1 ± 0.0 | 0.1 ± 0.0 | 0.02 (0.00-11.80, p=.229) |  |
| Average Arterial Diameter (unit: μm) | Mean ± SD | 70.6 ± 8.7 | 69.9 ± 9.0 | 0.99 (0.97-1.01, p=.412) |  |
| Average Arterial Diameter between 0.5-1.0 PD | Mean ± SD | 80.0 ± 9.4 | 78.7 ± 9.9 | 0.99 (0.97-1.00, p=.155) |  |
| Average Arterial Diameter between 1.5-2.0 PD | Mean ± SD | 76.7 ± 9.7 | 75.1 ± 10.0 | 0.98 (0.97-1.00, p=.064) |  |
| Average Arterial Diameter between 2.0-2.5 PD | Mean ± SD | 73.0 ± 9.3 | 72.4 ± 10.2 | 0.99 (0.98-1.01, p=.476) |  |
| Arterial Diameter within 3mm of Fovea Centralis | Mean ± SD | 70.7 ± 9.8 | 70.6 ± 10.5 | 1.00 (0.98-1.02, p=.888) |  |
| Arterial Diameter within 5mm of Fovea Centralis | Mean ± SD | 57.2 ± 9.4 | 56.8 ± 9.9 | 1.00 (0.98-1.01, p=.616) |  |
| Arterial Diameter in the Superior Disk Region | Mean ± SD | 69.2 ± 8.8 | 68.7 ± 9.4 | 0.99 (0.98-1.01, p=.601) |  |
| Arterial Diameter in the Temporal Disk Region | Mean ± SD | 78.8 ± 9.8 | 78.1 ± 9.9 | 0.99 (0.98-1.01, p=.440) |  |
| Arterial Diameter in the Inferior Disk Region | Mean ± SD | 61.4 ± 7.7 | 60.5 ± 8.7 | 0.99 (0.97-1.01, p=.235) |  |
| Arterial Diameter in the Nasal Disk Region | Mean ± SD | 80.2 ± 10.3 | 79.3 ± 10.7 | 0.99 (0.98-1.01, p=.369) |  |
| Average Venous Diameter (unit: μm) | Mean ± SD | 59.4 ± 11.8 | 59.5 ± 12.2 | 1.00 (0.99-1.02, p=.891) |  |
| Average Venous Diameter between 0.5-1.0 PD | Mean ± SD | 70.6 ± 8.7 | 69.9 ± 9.0 | 0.99 (0.97-1.01, p=.412) |  |
| Average Venous Diameter between 1.0-1.5 PD | Mean ± SD | 105.0 ± 12.1 | 104.6 ± 13.6 | 1.00 (0.98-1.01, p=.699) |  |
| Average Venous Diameter between 1.5-2.0 PD | Mean ± SD | 102.1 ± 12.1 | 102.9 ± 12.9 | 1.00 (0.99-1.02, p=.496) |  |
| Average Venous Diameter between 2.0-2.5 PD | Mean ± SD | 99.8 ± 14.0 | 98.8 ± 14.7 | 1.00 (0.98-1.01, p=.422) |  |
| Venous Diameter within 3mm of Fovea Centralis | Mean ± SD | 95.2 ± 14.7 | 95.5 ± 15.9 | 1.00 (0.99-1.01, p=.865) |  |
| Venous Diameter within 5mm of Fovea Centralis | Mean ± SD | 59.8 ± 8.9 | 59.2 ± 9.8 | 0.99 (0.98-1.01, p=.475) |  |
| Venous Diameter in the Superior Disk Region | Mean ± SD | 85.0 ± 12.1 | 85.9 ± 13.1 | 1.01 (0.99-1.02, p=.432) |  |
| Venous Diameter in the Temporal Disk Region | Mean ± SD | 110.6 ± 13.9 | 108.5 ± 13.4 | 0.99 (0.98-1.00, p=.087) |  |
| Venous Diameter in the Inferior Disk Region | Mean ± SD | 73.3 ± 10.5 | 73.3 ± 11.4 | 1.00 (0.98-1.02, p=.942) |  |
| Venous Diameter in the Nasal Disk Region | Mean ± SD | 115.3 ± 14.9 | 114.5 ± 14.4 | 1.00 (0.98-1.01, p=.535) |  |
| Arterial to Venous Diameter Ratio | Mean ± SD | 89.2 ± 15.7 | 89.5 ± 17.1 | 1.00 (0.99-1.01, p=.827) |  |
| Angle between Vascular Arc and Fovea (unit: degrees) | Mean ± SD | 0.8 ± 0.1 | 0.8 ± 0.1 | 0.30 (0.04-2.56, p=.274) |  |
| Distance between Vascular Arc and Fovea Intersection (unit: μm) | Mean ± SD | 85.1 ± 7.5 | 84.9 ± 7.6 | 1.00 (0.97-1.02, p=.795) |  |
| Angle between Vascular Arc and 1PD Outside Disk (unit: degrees) | Mean ± SD | 9207.5 ± 1103.4 | 9091.1 ± 1033.3 | 1.00 (1.00-1.00, p=.219) |  |
| Distance between Vascular Arc and 1PD Outside Disk Intersection (unit: μm) | Mean ± SD | 129.3 ± 7.7 | 128.9 ± 7.4 | 0.99 (0.97-1.02, p=.574) |  |
| Angle between Vascular Arc and 2PD Outside Disk (unit:degree). | Mean ± SD | 3710.3 ± 291.3 | 3700.2 ± 278.8 | 1.00 (1.00-1.00, p=.691) |  |
| Distance between Vascular Arc and 2PD Outside Disk Intersection (unit: μm) | Mean ± SD | 111.0 ± 9.3 | 110.5 ± 9.0 | 0.99 (0.97-1.01, p=.524) |  |
| Optic Disk Area (unit: mm) | Mean ± SD | 6764.2 ± 548.0 | 6735.4 ± 522.4 | 1.00 (1.00-1.00, p=.544) |  |
| Horizontal Diameter of Optic Disk (unit: μm) | Mean ± SD | 2.8 ± 0.5 | 2.8 ± 0.5 | 0.95 (0.68-1.33, p=.748) |  |
| Vertical Diameter of Optic Disk (unit: μm) | Mean ± SD | 1789.0 ± 174.8 | 1793.8 ± 191.9 | 1.00 (1.00-1.00, p=.775) |  |
| Optic Disk Roundness | Mean ± SD | 1994.2 ± 198.2 | 1982.2 ± 185.3 | 1.00 (1.00-1.00, p=.478) |  |
| Optic Disk Tilt Angle (unit: degrees) | Mean ± SD | 0.8 ± 0.1 | 0.9 ± 0.1 | 1.70 (0.06-46.05, p=.752) |  |
| Distance from Center of Optic Disk to Boundary (unit: μm) | Mean ± SD | -7.4 ± 74.3 | -10.3 ± 72.8 | 1.00 (1.00-1.00, p=.663) |  |
| Distance from Optic Disk to Fovea (unit: μm) | Mean ± SD | 3097.1 ± 486.6 | 3102.5 ± 552.3 | 1.00 (1.00-1.00, p=.910) |  |
| Angle between Line Connecting Fovea and Optic Disk (unit: degrees) | Mean ± SD | 4996.8 ± 291.1 | 4953.3 ± 309.9 | 1.00 (1.00-1.00, p=.111) |  |
| Ratio of Long Axis to Short Axis of Optic Disk | Mean ± SD | 6.2 ± 3.6 | 6.1 ± 3.5 | 0.99 (0.95-1.04, p=.824) |  |
| Distance from Center of Fovea to Boundary (unit: μm) | Mean ± SD | 1.1 ± 0.1 | 1.1 ± 0.1 | 0.28 (0.02-3.13, p=.299) |  |
| Cup Area .unit. mm.. | Mean ± SD | 4031.7 ± 401.8 | 4033.9 ± 434.9 | 1.00 (1.00-1.00, p=.952) |  |
| Horizontal Diameter of Optic Cup (unit: μm) | Mean ± SD | 0.7 ± 0.3 | 0.7 ± 0.3 | 0.89 (0.50-1.59, p=.704) |  |
| Vertical Diameter of Optic Cup (unit: μm) | Mean ± SD | 926.2 ± 196.7 | 923.2 ± 203.7 | 1.00 (1.00-1.00, p=.869) |  |
| Optic Cup Roundness | Mean ± SD | 944.4 ± 194.7 | 934.4 ± 181.4 | 1.00 (1.00-1.00, p=.546) |  |
| Area Cup to Disk Ratio | Mean ± SD | 0.9 ± 0.0 | 0.9 ± 0.0 | 1.14 (0.02-70.09, p=.950) |  |
| Horizontal Cup to Disk Ratio | Mean ± SD | 0.3 ± 0.1 | 0.2 ± 0.1 | 0.70 (0.06-8.32, p=.775) |  |
| Vertical Cup to Disk Ratio | Mean ± SD | 0.5 ± 0.1 | 0.5 ± 0.1 | 0.58 (0.06-5.41, p=.630) |  |
| Disk Rim I Distance (unit: μm) | Mean ± SD | 0.5 ± 0.1 | 0.5 ± 0.1 | 0.78 (0.05-11.60, p=.854) |  |
| Disk Rim N Distance (unit: μm) | Mean ± SD | 525.8 ± 79.0 | 525.8 ± 76.2 | 1.00 (1.00-1.00, p=.995) |  |
| Disk Rim S Distance (unit: μm) | Mean ± SD | 409.8 ± 71.1 | 403.4 ± 70.9 | 1.00 (1.00-1.00, p=.317) |  |
| Disk Rim T Distance (unit: μm) | Mean ± SD | 527.2 ± 79.6 | 527.6 ± 83.5 | 1.00 (1.00-1.00, p=.963) |  |
| Vertical to Horizontal Ratio of Optic Disk Vascular Arc Direction | Mean ± SD | 450.7 ± 108.3 | 463.4 ± 112.8 | 1.00 (1.00-1.00, p=.203) |  |
| Optic Disk Short to Long Axis Ratio | Mean ± SD | 1.1 ± 0.1 | 1.1 ± 0.1 | 0.33 (0.04-2.77, p=.306) |  |
| Angle between Optic Disk Long Axis and Vertical Line | Mean ± SD | 0.9 ± 0.0 | 0.9 ± 0.1 | 8.12 (0.31-215.80, p=.211) |  |
| Maximum Rim Distance between Optic Disk and Optic Cup | Mean ± SD | -2.6 ± 24.1 | -2.5 ± 24.7 | 1.00 (0.99-1.01, p=.973) |  |
| Minimum Rim Distance between Optic Disk and Optic Cup | Mean ± SD | 1552.4 ± 186.9 | 1547.1 ± 178.9 | 1.00 (1.00-1.00, p=.743) |  |
| Minimum Rim Distance between Optic Disk and Optic Cup 1 | Mean ± SD | 353.4 ± 63.8 | 350.1 ± 62.0 | 1.00 (1.00-1.00, p=.559) |  |
| Angle between Minimum Rim of Optic Disk and Horizontal Line Start | Mean ± SD | -30.9 ± 107.4 | -31.4 ± 104.8 | 1.00 (1.00-1.00, p=.957) |  |
| Angle between Minimum Rim of Optic Disk and Horizontal Line End | Mean ± SD | -28.3 ± 108.5 | -31.4 ± 105.0 | 1.00 (1.00-1.00, p=.747) |  |
| Angle between Line Connecting Fovea and Optic Disk and Long Axis of Optic Disk | Mean ± SD | 68.0 ± 17.3 | 66.5 ± 17.2 | 0.99 (0.98-1.01, p=.311) |  |
| Arterial Centerline Fractal Dimension | Mean ± SD | 1.3 ± 0.0 | 1.3 ± 0.0 | 0.40 (0.01-15.58, p=.625) |  |
| Vascular Centerline Fractal Dimension | Mean ± SD | 1.4 ± 0.0 | 1.4 ± 0.0 | 0.05 (0.00-4.50, p=.194) |  |
| Venous Centerline Fractal Dimension | Mean ± SD | 1.3 ± 0.0 | 1.3 ± 0.0 | 0.01 (0.00-1.00, p=.050) | 245108861388.53 (0.02-3186040700930394712702976.00, p=.089) |
| Retinal Arterial Density | Mean ± SD | 0.0 ± 0.0 | 0.0 ± 0.0 | 0.00 (0.00-155.66, p=.120) |  |
| Retinal Venous Density | Mean ± SD | 0.1 ± 0.0 | 0.0 ± 0.0 | 0.00 (0.00-Inf, p=.983) |  |
| Arterial Fractal Dimension | Mean ± SD | 1.4 ± 0.0 | 1.4 ± 0.0 | 0.13 (0.00-10.71, p=.366) |  |
| Arterial Vessel Length | Mean ± SD | 763.3 ± 172.2 | 746.7 ± 186.4 | 1.00 (1.00-1.00, p=.308) |  |
| Venous Vessel Length | Mean ± SD | 654.1 ± 120.8 | 652.5 ± 136.2 | 1.00 (1.00-1.00, p=.889) |  |
| Main Arterial.Venous Ratio in Vascular Arc | Mean ± SD | 0.7 ± 0.1 | 0.7 ± 0.1 | 0.72 (0.14-3.76, p=.701) |  |
| Main Arterial.Venous Ratio in Lower Vascular Arc | Mean ± SD | 0.6 ± 0.1 | 0.6 ± 0.1 | 1.13 (0.24-5.19, p=.879) |  |
| Main Arterial.Venous Ratio in Upper Vascular Arc | Mean ± SD | 0.7 ± 0.1 | 0.7 ± 0.1 | 0.62 (0.12-3.20, p=.567) |  |
| Average Arterial Branching Angle | Mean ± SD | 64.5 ± 10.1 | 63.1 ± 10.5 | 0.99 (0.97-1.00, p=.124) |  |
| Average Venous Branching Angle | Mean ± SD | 64.1 ± 8.2 | 63.9 ± 8.5 | 1.00 (0.98-1.02, p=.850) |  |
| Distance from Nasal Edge of Optic Disk to Fovea Center (unit: μm) | Mean ± SD | 5883.8 ± 321.1 | 5839.2 ± 323.6 | 1.00 (1.00-1.00, p=.124) |  |
| Arterial Vessel Area | Mean ± SD | 4.2 ± 0.8 | 4.1 ± 0.8 | 0.85 (0.68-1.07, p=.161) |  |
| Vascular Area | Mean ± SD | 10.6 ± 1.1 | 9.7 ± 1.3 | 0.54 (0.46-0.65, p<.001) | 1.72 (0.79-3.75, p=.176) |
| Vascular Area within 0.5-1.0 PD | Mean ± SD | 1.4 ± 0.2 | 1.4 ± 0.2 | 0.47 (0.22-0.99, p=.048) | 0.66 (0.04-10.06, p=.768) |
| Vascular Area within 1.5-2.0 PD | Mean ± SD | 1.6 ± 0.3 | 1.5 ± 0.3 | 0.73 (0.41-1.31, p=.290) |  |
